# Dynamics of flavor compounds and microbial communities in sour cream with different fat contents

**DOI:** 10.1016/j.fochx.2026.103920

**Published:** 2026-04-25

**Authors:** Songlin Ma, Huanchang Zhang, Guojiao Wang, Xin Cai, Qing Hong, Zhenmin Liu

**Affiliations:** a State Key Laboratory of Dairy Biotechnology, Key Laboratory of Functional Dairy Products Processing, Ministry of Agriculture and Rural Affairs, Shanghai Engineering Research Center of Dairy Biotechnology, Dairy Research Institute, Bright Dairy & Food Co. Ltd., Shanghai 201103, China; b College of Food Science and Technology, Shanghai Ocean University, Shanghai 201306, China

**Keywords:** Sour cream, Fat content, Flavor compounds, Metabolomics, Microorganism

## Abstract

Milk fat is an important nutritional component in sour cream and contributes to its unique flavor. This study investigated the dynamic changes in flavor compounds and microbial communities of sour cream with different fat content. The results showed that fat content is a key factor affecting the flavor of sour cream. A total of 19 key aroma compounds were identified by GC–MS, including esters, methyl ketones, aliphatic aldehydes, lactones, and sulfur compounds. These substances are mainly derived from esterification, amino acid metabolism, and β-oxidation of fatty acids. Additionally, *Lactococcus* was the dominant microorganism in sour cream and was closely associated with the formation of various flavor compounds. Notably, the F20 group (20% fat) exhibited superior sensory properties and flavor characteristics. These findings lay a theoretical foundation for optimizing sour cream quality and enhancing flavor.

## 1 Introduction

Sour cream, a high-fat dairy product obtained by fermentation of fresh cream with lactic acid bacteria (Kim et al., 2021), is widely used in baked goods, dips, and other culinary applications due to its rich flavor and smooth texture (El-Sayed et al., 2024). Nutritionally, sour cream is rich in milk fat, as well as proteins, vitamins, and minerals. Notably, as a critical constituent, milk fat significantly affects the flavor and sensory quality of fermented dairy products (Chowdhury et al., 2024). Previous studies have reported that high-fat Greek yogurt exhibits a more pronounced creamy flavor and a denser texture, thereby enhancing its viscosity and overall sensory scores (Desai et al., 2013). The addition of milk fat can influence the growth and metabolic activities of *Lactobacillus casei* GBHM-21, stimulating the production of lipase, protease, and aminotransferase, which in turn promotes the generation of flavor compounds during fermentation (Bao et al., 2016). Additionally, increased milk fat content leads to higher concentrations of ketone compounds (such as 2-pentanone, 2-heptanone, and 3-hydroxy-2-butanone) in fermented milk (Tan et al., 2024). However, the impact of fat content on sour cream production remains understudied, particularly in terms of flavor precursor composition, flavor development, and microbial dynamics.

Numerous studies have demonstrated that the sensory characteristics of fermented dairy products, including aroma, flavor, and texture, significantly influence consumer preferences and acceptance (Penland et al., 2021). These characteristics are predominantly governed by volatile organic compounds (VOCs) generated during fermentation and ripening. For instance, the accumulation of 3-methylbutanal in fermented milk imparts a characteristic caramel flavor (Xia, Yang, et al., 2023). Moreover, Wang et al. (2023) identified volatile flavor compounds in sour cream, including hexanoic acid, 1-octen-3-one, 2-heptanol, and ethyl acetate, which contribute fatty, creamy, and fermented notes. Currently, HS-SPME-GC–MS is a widely applied technique for the separation and identification of volatile compounds in food (Parastar & Weller, 2024). Esmaeilzadeh et al. (2021) employed HS-SPME-GC–MS to analyze volatile compounds in Kope cheese, such as butanoic acid, 2,3-butanediol, ethyl hexanoate, 2-heptanone, and diacetyl. Similarly, (*E*)-2-octen-1-ol, 1-octen-3-ol, 2-nonanone, 2,3-butanedione, and 2-heptanol have been identified as key volatile compounds in cream fermented by *Lactiplantibacillus plantarum* B22 (Chai et al., 2025).

Meanwhile, lactic acid bacteria (LAB) fermentation plays a crucial role in developing the flavor characteristics of sour cream (Feng et al., 2025). During fermentation, enzymes secreted by LAB (such as proteases, lipases, and esterases) facilitate proteolysis and lipolysis, forming small-molecule peptides, free amino acids (FAAs), and free fatty acids (FFAs) (Tian et al., 2023). These are key precursors for the biosynthesis of various flavor compounds. Among these, small peptides and amino acids contribute to umami, sweet, and bitter tastes. Volatile fatty acids (including butyric, caprylic, and nonanoic acids) impart sour, fruity, and fatty notes to the product (Wang, Sun, et al., 2024). In contrast, long-chain fatty acids (namely oleic, linoleic, and linolenic acids) are involved in flavor development but may also generate soapy or bitter off-flavors (Omar et al., 2016). Furthermore, LAB can further convert FAAs, FFAs, and lactose into various flavor compounds, including organic acids, aliphatic aldehydes, methyl ketones, lactones, aromatic compounds, and sulfur-containing compounds (Vélez et al., 2017; Xiang et al., 2023). Ultimately, these substances enhance the nutritional value of the product while imparting its unique flavor and texture (Borges et al., 2019). In kefir samples, carboxylic acids, methyl ketones, and aromatic alcohols were identified as the predominant volatile compounds, which are closely associated with the metabolic activities of bacteria and yeasts (Dertli & Çon, 2017). Wang, Wang, et al. (2024) demonstrated that *Lactococcus lactis*, *Trichococcus*, and *Monascus* were significantly correlated with key aroma compounds, including hexanoic acid, valeric acid, and 2-pentanone in *Monascus* cheese, as identified by GC–MS and high-throughput sequencing. However, research on the correlation between microorganisms and flavor compounds in sour cream has not yet been reported.

In summary, previous studies have primarily focused on sour cream with a uniform fat content, overlooking the crucial role of varying fat levels in shaping microbial community dynamics and the release of volatile compounds. Besides, a systematic understanding of flavor formation in sour cream remains elusive. Therefore, this study aims to systematically investigate the differences in physicochemical properties, sensory characteristics, flavor precursors, volatile compounds, and microbial composition of sour cream with varying fat contents. The volatile organic compound profiles of sour cream were determined using HS-SPME-GC–MS, and the compositions of FAAs and FFAs were analyzed. Concurrently, amplicon sequencing was employed to characterize the dynamic changes in the microbial community and to elucidate its potential correlations with key flavor compounds. These findings will not only broaden and deepen the current knowledge base but also provide a scientific basis for the dairy industry to develop sour cream products with enhanced quality and superior flavor.

## 2 Materials and methods

### 2.1 Materials

Raw milk (3.5% fat, 3.2% protein) and fresh cream (separated from milk, 40% fat, 1.8% protein) were obtained from Bright Dairy & Food Co., Ltd. The commercial starter culture CHOOZIT MM 100 (*Lactococcus lactis* subsp. *lactis*、*Lactococcus cremoris* and *Lactococcus lactis* subsp. *lactis* biovar. *diacetylactis*) was provided by Danisco (China) Ltd.

### 2.2 Samples preparation and collection

The preparation process of sour cream involved: cream was standardized to fat contents of 10%, 20%, 30%, and 40% (*w/w*), designated as F10, F20, F30, and F40, respectively. The cream was homogenized at 60–65 °C and 5–7 MPa, pasteurized at 90 °C for 5 min, and cooled to 28–30 °C before starter culture inoculation. Fermentation was conducted at 30 °C until reaching a pH of 4.60 ± 0.02, followed by curd disruption. The product was subsequently matured at 4 °C for 12 h to obtain the final sour cream. Samples were collected at three stages: unfermented (A), post-fermentation (B), and post-maturation (C). All samples were stored at −80 °C until further analysis.

### 2.3 Measurements of physicochemical parameters

The protein, fat, carbohydrate, ash, and total solids contents were determined in accordance with Chinese National Standards GB 5009.5–2016, GB 5009.6–2016, GB 28050–2011, GB 5009.4–2016, and GB 5009.3–2016. Non-fat milk solid content was calculated by deducting fat content from the total solids content.

### 2.4 Sensory evaluation

The sensory evaluation was conducted in a food laboratory. The sensory evaluation panel consisted of 10 assessors (4 males, 6 females, aged 20–35) who had received professional training in sensory evaluation. Verbal informed consent was obtained from all participants prior to their involvement in the study, and their rights and privacy were protected throughout the research process. Before evaluation, all samples were coded with three-digit numbers and presented to the panelists randomly at room temperature. Quality attributes of the samples, including color, texture, aroma, taste, and overall acceptability, were evaluated using a 9-point scale (0 = not perceivable, 9 = strongly perceivable) (Hu et al., 2025).

### 2.5 Volatile flavor compounds analysis

The analysis of flavor compounds in sour cream samples at different stages was performed with reference to the method reported by Zhang, Zheng, et al. (2025), with slight modifications. Briefly, 5 g of the sample was transferred to a 20 mL headspace vial and incubated at 80 °C for 10 min. A DVB/CAR/PDMS fiber (50/30 μm, 2 cm, Supelco Inc., Bellefonte, PA, USA) was exposed to the headspace for extraction at 80 °C for 30 min. Subsequently, the fiber was desorbed in the GC injector for 5 min. Volatile compounds were separated and identified using a gas chromatography-time-of-flight mass spectrometry (GC-TOFMS) system (7890B GC, Agilent Technologies Inc., California, USA; Pegasus BT MS, LECO, USA) equipped with a DB-WAX capillary column (30 m × 0.25 mm × 0.25 μm, Agilent Technologies Inc., California, USA). Helium (He) was used as the carrier gas at a flow rate of 1.0 mL/min. The injector temperature was maintained at 245 °C, while the ion source temperature was set at 220 °C. The GC temperature program was started at 40 °C for 3 min, then increased to 230 °C at a rate of 10 °C/min, and held for 6 min. The MS system was equipped with an electron impact ionization (EI) source at 70 eV, with a mass scan range of 35–450 *m*/*z* and a scan frequency of 15 spectra/s. Raw data were processed using ChromaTOF software (v4.71, LECO) for baseline denoising, smoothing, peak extraction, deconvolution, and peak alignment. Retention index (RI) was calculated based on n-alkane standards (C7-C30), and compound identification was achieved by comparing their mass spectra and retention indices with standards in the NIST database. Data with a matching score above 700 were recorded. The relative contents of compounds were calculated using the peak area normalization method.

The contribution of volatile compounds to the overall flavor profile is evaluated using the relative odor activity value (ROAV), with the formula as follows:

ROAV_B_ = 100 × (*Peak*_*B*_/*T*_*B*_ × *T*_*A*_/*Peak*_*A*_).

Note: *T*_*A*_ and *Peak*_*A*_ are assumed to be the odor threshold and peak area, respectively, of the compound with the lowest odor threshold in the sample. *T*_*B*_ and *Peak*_*B*_ are assumed to be the odor threshold and peak area of the compounds to be measured. The ROAV for component A was set to a standard value of 100.

### 2.6 Determination of free amino acid composition

Free amino acid analysis was performed following the method of Zhao et al. (2025) with minor modifications. Briefly, 20 mg of the lyophilized sample was mixed with 1 mL of pre-cooled acetonitrile-methanol-water solution (2:2:1, *v/v*, containing isotopically labeled internal standards). The mixture was sonicated in an ice-water bath for 10 min and stored at −40 °C for 1 h to precipitate proteins. Subsequently, the mixture was centrifuged at 12,000 rpm for 15 min at 4 °C, and 100 μL of supernatant was collected directly for ultra-performance liquid chromatography-mass spectrometry (UPLC-MS) analysis.

Qualitative and quantitative analysis of FAAs was conducted using an Agilent 1290 Infinity series UPLC system coupled with a 6460 Triple Quadrupole mass spectrometer. Separation of compounds was performed using an ACQUITY BEH Amide column (100 × 2.1 mm, 1.7 μm, Waters, MA, USA). The mobile phase consisted of 1% formic acid in aqueous solution (A) and 1% formic acid in acetonitrile (B), with a column temperature of 35 °C and an injection volume of 1 μL. The elution program was: 0–0.5 min 10% B; 0.5–6.5 min 10%–30% B; 9.5–10 min 50%–95% B; 10–12 min 95% B; 12–12.1 min 95%–10% B; 12.1–15 min 10% B.

Mass spectrometry analysis was performed in positive ion mode using electrospray ionization (ESI). The ion source parameters are set as follows: Capillary voltage = 4000 V, gas (N_2_) temperature = 300 °C, gas (N_2_) flow = 5 L/min, sheath gas (N_2_) temperature = 250 °C, sheath gas flow = 11 L/min, nebulizer = 45 psi. The multiple reaction monitoring (MRM) mode was used to acquire mass spectrometric data.

### 2.7 Determination of free fatty acid composition

Free fatty acids were extracted following the method of Xu et al. (2020). The analysis was performed using GC–MS (7890B—5977B, Agilent, USA) equipped with a DB-FastFAME column (90 m × 250 μm × 0.25 μm). The internal standard was Stearic-d35 acid (98%; SIGMA, USA). Specifically, 25 mg of sour cream was mixed with 1 mL of extraction solvent (isopropanol: n-hexane, 2:3, *v/v*), and the mixture was sonicated in an ice-water bath for 15 min. The sample was centrifuged at 12,000 rpm for 15 min at 4 °C, and the supernatant was collected. After evaporation to dryness under a nitrogen stream, 500 μL of methanol: trimethylsilyl diazomethane (2moL/L in Hexane, T903597, Shanghai Macklin Biochemical Co., Ltd., China) solution (1:2, *v/v*) was added. Following a 30-min methylation reaction, the mixture was dried under nitrogen. The residue was reconstituted in n-hexane, centrifuged at 12,000 rpm for 1 min, and the supernatant was subjected to GC–MS analysis. Each sample was injected at a volume of 1 μL with a split mode (5:1). Helium was used as the carrier gas at a flow rate of 3 mL/min and a column head pressure of 46 psi. The column temperature program was as follows: initial temperature 75 °C, increased to 200 °C at 50 °C/min (held for 15 min), then to 210 °C at 2 °C/min (held for 1 min), and finally to 230 °C at 10 °C/min (held for 15 min). The injector and ion source temperatures were maintained at 230 °C. The energy was 70 eV in electron impact mode. Quantification of FFAs is expressed as mg/kg.

### 2.8 Microbial community analysis

Genomic DNA was extracted from all samples using the OMEGA DNA Kit (Omega, D5625–01, Norcross, GA, USA). The V3-V4 hypervariable region of the bacterial 16S rRNA gene was amplified by PCR with specific primers 338F (5’-ACTCCTACGGGAGGCAGCA-3′) and 806R (5′-GGACTACHVGGGTWTCTAAT-3′) (Wang et al., 2022). DNA concentration and purity were quantified using a Microplate reader (BioTek, FLx800) and assessed by 2% agarose gel electrophoresis, respectively. Library preparation was conducted using the Illumina TruSeq Nano DNA LT Library Prep Kit. The constructed libraries were quality-controlled using an Agilent Bioanalyzer 2100 and Promega QuantiFluor. Paired-end sequencing was conducted on the Illumina NovaSeq 6000 (Illumina, San Diego, USA) platform. Amplicon sequence variants (ASVs) were obtained after quality filtering, denoising, and chimera removal using QIIME 2. The 16S rRNA gene sequences were annotated and classified against the Silva database. Alpha diversity indices, including *Chao1*, *Simpson*, and *Shannon* indices, were calculated concurrently.

### 2.9 Statistical analysis

All experiments were conducted in triplicate, and data were presented as mean ± standard deviation (SD). Statistical analysis was assessed using one-way ANOVA with Duncan multiple comparison post hoc tests via SPSS 30.0.0 software (IBM Corp., Armonk, NY, USA). Partial least squares-discriminant analysis (PLS-DA) and variable importance in projection (VIP) values were identified through MetaboAnalyst 6.0 (https://www.metaboanalyst.ca). Heatmaps were visualized with TBtools-II software (Toolbox for Biologists, version 2.357, China) (Chen et al., 2023). Spearman correlation analysis were conducted using OmicStudio tools (https://www.omicstudio.cn/tool). Pathway enrichment analysis was performed based on the KEGG database.

## 3 Results and discussion

### 3.1 Changes in physicochemical properties

The physicochemical indicators of sour cream with different fat contents are presented in Table 1. The protein content of the sour cream decreased significantly with increasing fat content (*P* < 0.05), with values of 2.90 ± 0.03, 2.65 ± 0.06, 2.31 ± 0.01, and 1.97 ± 0.02 g/100 g, respectively. Conversely, the total solid content showed a progressive increase, ranging from 18.38 ± 0.09 to 47.11 ± 0.09 g/100 g. Lactose, the predominant carbohydrate in milk, acts as a fermentation substrate and contributes to the nutritional and flavor of dairy products. The F10 group exhibited a significantly higher carbohydrate content (4.61 ± 0.14 g/100 g) compared to the other groups (*P* < 0.05). Non-fat milk solids serve as a comprehensive nutritional parameter reflecting the levels of protein, lactose, and minerals (Bassbasi et al., 2014). Notably, the non-fat solid content was highest in the F10 group (8.10 ± 0.12 g/100 g) and lowest in the F30 group (5.18 ± 0.04 g/100 g). These results indicate that the sour cream is nutrient-rich, and the observed variations among groups were primarily attributed to the standardization of raw milk.

**Table 1:** Physicochemical indicators and sensory properties of sour cream.

Parameters	F10	F20	F30	F40
Protein (g/100 g)	2.90 ± 0.03^a^	2.65 ± 0.06^b^	2.31 ± 0.01^c^	1.97 ± 0.02^d^
Fat (g/100 g)	10.28 ± 0.03^d^	20.10 ± 0.03^c^	30.49 ± 0.02^b^	40.60 ± 0.12^a^
Carbohydrate (g/100 g)	4.61 ± 0.14^a^	3.64 ± 0.15^c^	2.41 ± 0.03^d^	4.19 ± 0.19^b^
Ash (g/100 g)	0.59 ± 0.01^a^	0.54 ± 0.01^b^	0.46 ± 0.01^c^	0.35 ± 0.01^d^
Total solids (g/100 g)	18.38 ± 0.09^d^	26.93 ± 0.24^c^	35.67 ± 0.03^b^	47.11 ± 0.09^a^
Non-fat milk solid (g/100 g)	8.10 ± 0.12^a^	6.83 ± 0.21^b^	5.18 ± 0.04^d^	6.51 ± 0.18^c^
Color	7.50 ± 0.71^ab^	8.00 ± 0.82^a^	7.00 ± 0.82^b^	6.10 ± 0.74^c^
Texture	4.50 ± 0.85^c^	7.70 ± 0.95^a^	6.70 ± 0.95^b^	6.30 ± 0.95^b^
Aroma	7.00 ± 0.94^ab^	7.50 ± 0.53^a^	6.40 ± 0.84^bc^	5.70 ± 0.95^c^
Taste	6.00 ± 0.82^c^	7.00 ± 0.94^a^	6.90 ± 0.88^ab^	6.30 ± 1.16^ab^
Overall acceptability	6.00 ± 0.94^bc^	7.60 ± 0.84^a^	6.80 ± 0.79^ab^	5.40 ± 1.07^c^

Note: All the values are Mean ± Standard Deviation.

^abc^^d^ Mean values in the same row with different superscript letters are significantly different at 5% level of significance (*P* < 0.05).

### 3.2 Sensory analysis

Sensory properties are critical determinants of consumer preference. As presented in Table 1, the F10 group exhibited a softer texture (4.50) with the lowest taste score (6.00). Conversely, F30 and F40 samples exhibited a firmer texture with noticeable graininess. Among all samples, the F40 group received significantly lower color (6.10) and aroma (5.70) scores relative to the other samples, which markedly compromised its overall acceptability (5.40). This observation may be attributed to lipid oxidation, which reduced the lightness of the samples and led to the development of off-flavors (Dhakal et al., 2024). Notably, the F20 group received higher sensory scores in terms of color, texture, aroma, taste, and overall acceptability. Therefore, fat content has a significant effect on the sensory properties of sour cream, with the F20 group demonstrating the most desirable sensory quality.

### 3.3 Analysis of volatile flavor compounds in sour cream

**Fig. 1:**
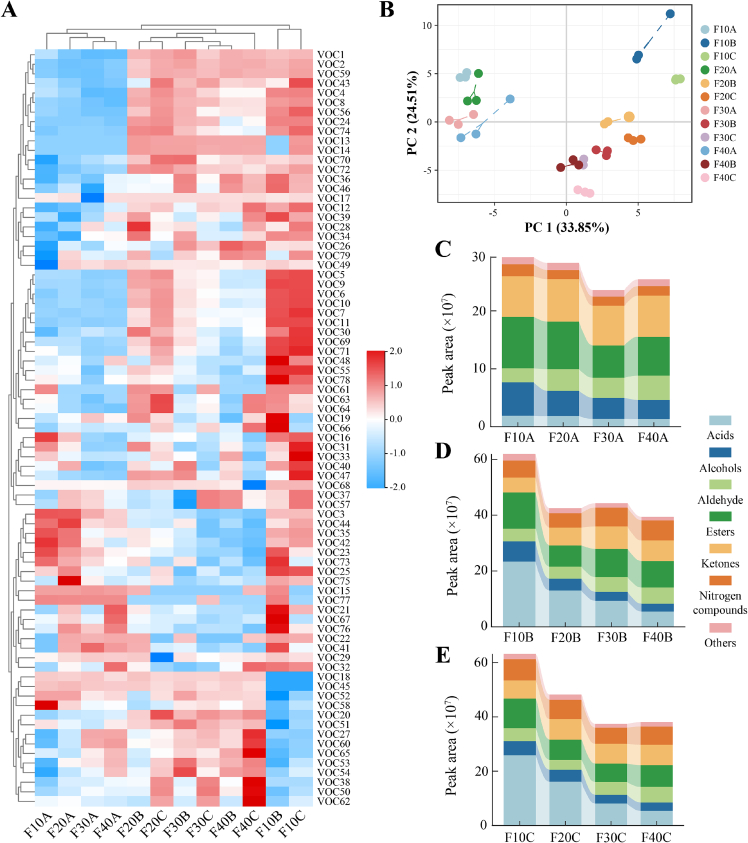
The variations in volatile compounds of sour cream. (A) The cluster heatmap of volatile compounds. (B) Principal components analysis score plot. (C), (D) and (E) Peak area of various volatile compounds.

To elucidate the variation patterns of flavor compounds during the production of sour cream with different fat contents, volatile flavor substances in the samples were identified using HS-SPME-GC-TOFMS. A total of 79 flavor compounds were detected, comprising 11 acids, 14 alcohols, 9 aldehydes, 14 esters, 8 ketones, 5 nitrogen compounds, 3 lactones, 3 sulfides, 7 hydrocarbons, 3 benzenes, and 2 halogenated compounds (Table S1). The cluster heatmap of volatile flavor compounds is shown in Fig. 1A, indicating that the content of flavor compounds significantly increased over time. In the principal components analysis (PCA) score plot, unfermented samples clustered on the left side, whereas fermented samples were predominantly distributed on the right (Fig. 1B), indicating that fermentation substantially influences the flavor profile in sour cream. Furthermore, partial overlap was observed between F30C and F40B, suggesting similar flavor characteristics, while significant differences existed among the remaining samples.

The changes in the types and contents of flavor compounds in sour cream with varying fat contents are presented in Fig. 1C-E. During fermentation and maturation, the content of acids gradually increases, imparting a rich sour flavor to the product. For instance, in the F10 group, the content of octanoic acid increased rapidly from 3.28% to 23.01%. Notably, the distribution of VOCs was significantly influenced by fat content. Increasing fat content reduced the concentrations of acids and alcohols, whereas the contents of aldehydes, esters, and nitrogen compounds progressively increased. Moreover, the total peak area of volatile flavor compounds gradually decreased, which may be attributed to the effect of increased fat content on the release of flavor compounds and their perception (Jo et al., 2018).

Volatile acids are primarily generated through lipolysis, along with the metabolism of lactose and amino acids, imparting the characteristic sour taste to sour cream. Furthermore, carboxylic acids serve as precursors for aldehydes, ketones, and esters, indirectly promoting the development of diverse flavors (Xu et al., 2025). Among the fermented samples, the F10 group exhibited the highest relative content of acids (37.80–40.95%). Compared to the F10 group, the acid content in the F40 group decreased significantly by approximately 65% (*P* < 0.05), indicating that milk fat content influences the release of acidic compounds. Octanoic acid was the most abundant acid, followed by 3-methylvaleric acid and nonanoic acid. Consistent findings have been reported by Beltrán-Barrientos et al. (2019) in yogurt. 2-Methylhexanoic acid and 4-methylnonanoic acid are associated with flavors characteristic of mutton. Long-chain fatty acids such as heptanoic, nonanoic, and decanoic acids possess high odor thresholds and contribute minimally to the overall flavor profile of sour cream. Only two acids with ROAV ≥1 (octanoic acid and 3-methylpentanoic acid) were present across all groups, generating pronounced sour and fatty notes.

Alcohols are produced through the oxidation of linolenic acid and 16-hydroperoxides, along with the reduction of carbonyl compounds (Li, Oey, & Kebede, 2024), which contributes to desirable flavor and aroma. These compounds typically exhibit high odor thresholds and influence flavor only at elevated concentrations (Xu et al., 2025). Among the 14 alcohols detected, 3-methyl-1-heptanol was the most abundant, constituting approximately 70% of the total alcohols. The concentration of alcohols demonstrated a decreasing trend with increasing milk fat content, potentially attributed to oxidation to aldehydes or esterification reactions. 3-Methyl-2-butanol exhibits fresh fruity notes, 1-octanol imparts mushroom and fatty characteristics, whereas 2-octen-1-ol and 2-hexen-1-ol contribute floral aromas. 1-Heptanol (green, nutty) and 1-octanol (floral) are recognized as common volatile flavor compounds with low odor thresholds in dairy products. Among the alcohols, 1-octen-3-ol was the sole odor-active compound that made a significant contribution to the overall flavor profile (ROAV >1). It originates from the degradation of polyunsaturated fatty acids catalyzed by lipoxygenase or the reduction of carbonyl compounds, imparting mushroom and green notes.

Aldehydes, characterized by low odor thresholds and high volatility, make significant contributions to the flavor profile of sour cream. They primarily originate from lipid oxidation and Strecker degradation of amino acids (Ping et al., 2024), imparting complex aroma characteristics to the product. However, elevated concentrations of aldehydes may lead to off-flavors (Wu et al., 2025). Unsaturated fatty acids (such as linoleic acid and linolenic acid) are oxidized to hydroperoxides under the action of enzymes or free radicals. Further oxidation forms small-molecule compounds, including heptanal, hexanal, and unsaturated aldehydes (Ghelichi et al., 2023). It can lead to the formation of rancid or oxidized off-flavors (Chen et al., 2017). The higher the fat content, the higher the degree of lipid oxidation (Bao and Pignitter, 2023). The F40 group exhibited the highest relative content of aldehydes (15.42%), which may adversely affect the sensory attributes. Furthermore, the reduced aldehyde content in the F10 group diminished the perception of fatty flavors. Among the aldehydes, five compounds exhibited ROAV >1: namely, pentanal, nonanal, decanal, 2-heptenal, and 2-octenal. Pentanal contributes fruity and fermented notes, nonanal is described as fresh and orange-like, and decanal imparts citrus peel-like aromas. 2-Heptenal and 2-octenal exhibit low concentrations but possess high ROAV values, imparting a green, fresh, and fatty aroma (Zhang, Dong, et al., 2025).

Esters, characteristic flavor components in milk, originate from esterification reactions between organic acids (or fatty acids) and alcohols, as well as microbial enzymatic processes (Que et al., 2023). These compounds can interact with alcohols and aldehydes, which mask the pungent odors of fatty acids and amines and the bitterness of amino acids, thereby positively contributing to sensory quality (Chi et al., 2022). During maturation, esters are hydrolyzed into acids and alcohols by microbial esterases or are converted to ketones via β-oxidation, leading to decreased concentrations. Propyl lactate, formed through esterification of lactic acid and propanol, was the most abundant ester and enhanced milky and fruity notes. Ethyl formate, an aromatic compound generated during fermentation, imparts a fresh, green, creamy aroma. Butyl acetate was identified as a key odorant in donkey milk (Li, Sun, et al., 2024). Methyl nonanoate and methyl decanoate are secondary metabolites produced by microorganisms. Three esters with ROAV >1 were identified, namely methyl butyrate, ethyl propionate, and methyl 4-methylpentanoate. These compounds predominantly exist in low-fat unfermented samples, imparting sweet and wine-like notes.

Ketones are primarily formed through the oxidation of lipids or the degradation of amino acids and have an important influence on the flavor profile of sour cream (Guo et al., 2022). During fermentation, ketones are further reduced to alcohols by reductases, thereby reducing their concentrations. In F40 sour cream, the levels of 2-octanone and 2-nonanone were significantly higher than in other groups. Capozzi et al. (2020) identified ketones as the predominant compounds in Mascarpone cheese, including 2-pentanone, 2-heptanone, and 2-nonanone. The biosynthesis of methyl ketones reportedly originates from β-oxidation of even-chain saturated fatty acids and decarboxylation of β-keto acids (Zhang et al., 2021), and they serve as indicators for assessing the oxidation degree of dairy products. Additionally, lipolysis during milk heat treatment accelerates methyl ketone formation. Methyl heptenone, described as “green, vegetable, and apple,” significantly enhances the sweetness intensity of fructose solutions (Dai et al., 2025). Acetophenone, a common volatile flavor compound in surface-ripened cheeses, is characterized by floral and woody notes (Bertuzzi et al., 2018). 2-Octanone, 2-nonanone, and 2-decanone are key aromatic compounds in sour cream (ROAV >1), produced via the pyruvate metabolic pathway and contributing desirable sweaty, cheesy, and floral. 6-Methyl-2-heptanone is derived from the oxidation of unsaturated fatty acids, serves as a potential indicator for evaluating tilapia freshness (Karbsri et al., 2024).

Lactones are formed via intramolecular esterification of hydroxy fatty acids within triglycerides and are key flavor compounds in dairy products. Chen et al. (2022) identified γ- and δ-lactones as prevalent lactones in cheeses (e.g., Cheddar, Gouda, and Parmesan), noting that their concentrations significantly increased with extended maturation time. Three lactone compounds were detected across all samples. γ-Lactones are derived from 4-hydroxy fatty acids, while δ-nonalactone is derived from 5-hydroxynonanoic acid via cyclase activity (Xing et al., 2025), imparting the sweet and milky notes. γ-Octanoic lactone and δ-octalactone (ROAV >1), exhibiting coconut and creamy aromas, enhance the milky perception of dairy products. These two lactones contribute positively to the overall flavor development of sour cream. Sulfides are typically generated from methanethiol produced via Strecker degradation of sulfur-containing amino acids (methionine) (Que et al., 2023), possess low sensory thresholds, and can cause off-flavors. For instance, dimethyl disulfide and dimethyl trisulfide, characterized by typical sulfurous, onion-like, and rotten egg odors, are identified as contributors to the undesirable flavors in sour cream.

**Fig. 2:**
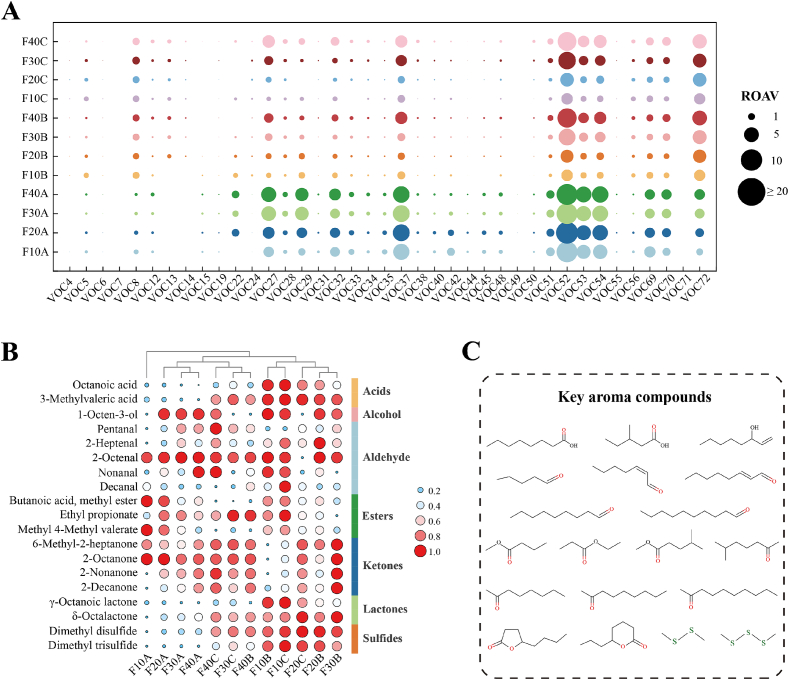
Identification of key aroma compounds of sour cream. (A) The ROAV of volatile compounds. (B) The bubble heatmap of aroma-contributing volatile compounds (ROAV >1). (C) The key aroma compounds of sour cream.

The ROAV was used to evaluate the contribution of flavor compounds to the overall flavor profile of sour cream, in which a higher ROAV indicates a greater contribution. Table S2 and Fig. 2A present the odor descriptors and relative odor activity values of the identified flavor compounds. A total of 40 flavor-active substances were identified in the sour cream samples, which imparted rich aromatic characteristics to the product. As illustrated in the bubble heatmap of relative concentrations (Fig. 2B), 19 aroma compounds were identified as key aroma compounds (ROAV ≥1). These compounds comprised five aldehydes, four ketones, three esters, two acids, one alcohol, two lactones, and two sulfides. Among these, ethyl propionate, 2-octanone, 2-nonanone, 2-decanone, γ-octanoic lactone, δ-octalactone, dimethyl disulfide, and dimethyl trisulfide were common key aroma compounds found in all groups, thus constituting the core flavor profile of the sour cream. This is likely attributable to the use of identical raw materials, preparation processes, and starter cultures (Wei et al., 2024). Additionally, ten compounds were found to play a significant supporting role in the overall flavor (0.1 ≤ ROAV ≤1). These included 1-heptanol, 2-octen-1-ol, 2-ethylhexanol, 2,4-dimethylbenzaldehyde (almond), 2,4-octadienal (melon), butyl acetate (banana), methyl nonanoate, methyl decanoate, 2-methyl-3-pentanone (mint), and acetophenone. The presence of these compounds enriched the flavor profile and enhanced the sensory characteristics of the sour cream.

In a word, the flavor of sour cream originates from the complex interactions among multiple volatile compounds. Aldehydes, ketones, esters, carboxylic acids, lactones, and sulfides were identified as the primary VOCs in sour cream, among which ketones, aldehydes, esters, and sulfides contributed most significantly to the overall flavor development. Meanwhile, fat content had a significant impact on the flavor profile of sour cream. The elevated levels of acids in the F10 group may have introduced undesirable sour off-notes, thereby affecting the sensory quality. The F30 and F40 groups exhibited pronounced fatty and creamy notes; however, the accumulation of aldehydes could lead to the generation of off-flavors, such as oxidized and rancid notes. In contrast, the F20 group stood out for its balanced profile of sour, sweet, and fruity aromas, accompanied by a lower abundance of off-flavor compounds.

### 3.4 Microbial community analysis of sour cream

**Fig. 3:**
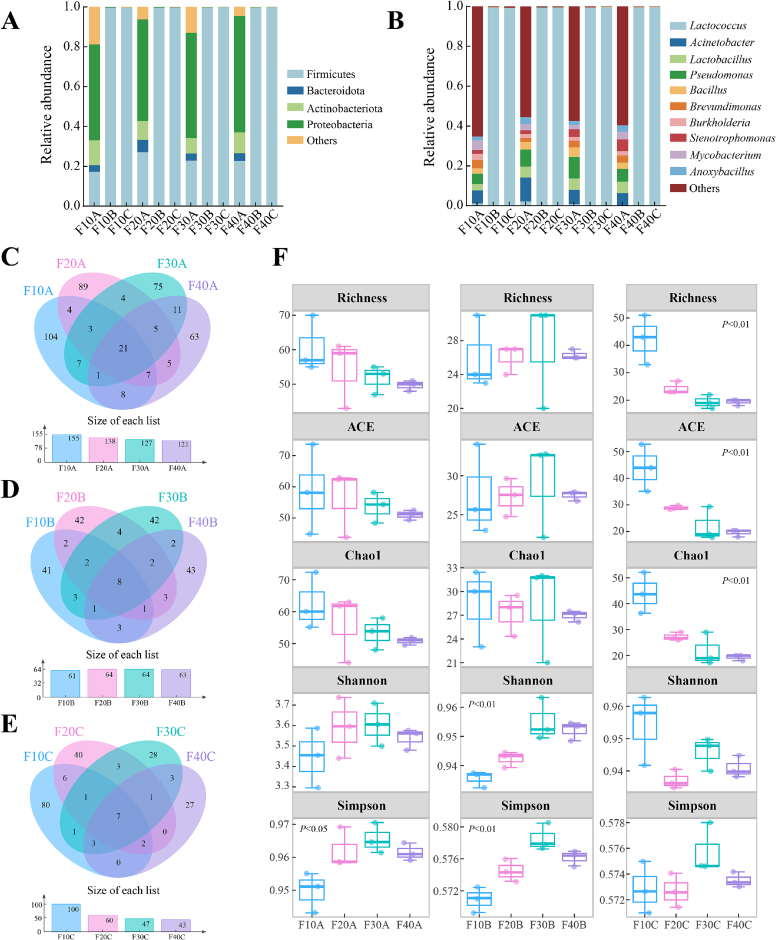
Changes of microbial community composition in sour cream. (A) Relative abundance of bacterial community at the phyla level. (B) Relative abundance of bacterial community at the genus level. (C), (D) and (E) The Venn diagram revealed the unique ASVs and shared ASVs across the different samples. (F) Changes of microbial diversity index.

Microorganisms enhance the flavor and nutritional properties of sour cream by producing a rich array of bioactive substances through their involvement in proteolysis, lipolysis, and carbohydrate metabolism. A thorough understanding of the composition and dynamics of the microbial community in fermented dairy products is crucial for improving their flavor and sensory quality. Fig. 3A shows the dynamic changes in the microbial composition of sour cream at the phylum level. Firmicutes, Proteobacteria, Actinobacteria, and Bacteroidota were the dominant phyla in the unfermented samples. As the fat content increased, the relative abundance of Proteobacteria gradually increased (from 48.19% to 58.49%), whereas Firmicutes and Actinobacteria showed an initial increase followed by a decrease. This may be attributed to Proteobacteria secreting highly active lipases to accelerate milk fat hydrolysis and sustain their growth and reproduction (Li, Ma, et al., 2025). Concurrently, as fermentation progressed, the abundance of Firmicutes increased significantly (from 17.20% to 99.57%), while that of Proteobacteria decreased significantly (from 58.49% to 0.12%). Other bacteria maintained a low abundance (< 0.5%). Firmicutes, which possess excellent acid tolerance, promote their growth and reproduction through protein and carbohydrate metabolism, leading to their continuous accumulation during fermentation (Zhu et al., 2024). However, the decline in Proteobacteria abundance can be attributed to the inhibition of their growth and reproduction under high-acidity conditions.

At the genus level, *Acinetobacter*, *Lactobacillus*, *Pseudomonas*, and other genera were mainly present in the unfermented samples (Fig. 3B). These microorganisms were mainly derived from raw milk or the processing environment. In contrast, *Lactococcus* was the dominant genus in the starter culture, maintaining a relative abundance of over 99% throughout the fermentation process. Its rapid growth led to a substantial accumulation of organic acids, creating a low-pH environment that effectively inhibited the growth of other microorganisms. A recent study found that *Lactococcus* was also the predominant microorganism in traditional sour creams, such as Buryatia (Russia) and Stake (Greece) (Papadimitriou et al., 2024). *Lactococcus* produces volatile flavor compounds through the protein proteolysis and amino acid metabolism pathways, playing an important role in the flavor formation of fermented milk and cheese (Xia, Yang, et al., 2023).

The Venn diagram illustrates the composition of ASV numbers across different samples, reflecting their similarities and differences. A total of 723 bacterial ASVs were detected across the 12 sample groups. The total number of ASVs in samples F10, F20, F30, and F40 was 316, 262, 238, and 227, respectively (Fig. 3C-E). Throughout the fermentation and maturation process, the total ASV counts were 541, 252, and 250, respectively, while the number of shared ASVs was 21, 8, and 7, respectively. Meanwhile, the richness and diversity of the microbial communities were assessed using the Richness, ACE, Chao1, Shannon, and Simpson indices (Fig. 3F). The sequence coverage for all sour cream samples exceeded 98%, indicating that the sequencing data were sufficient to comprehensively characterize the microbial communities. In general, the Chao1 index estimates the richness of microbial community, the Shannon index represents the diversity of detected microbial community in samples, and the Simpson index reflects the evenness of species distribution in samples (Wang et al., 2022). The alpha diversity indices were highest in the unfermented samples, suggesting a rich microbial community that likely originated from the raw milk or the processing environment. As fermentation progressed, the microbial diversity significantly decreased. This was attributed to the reduction in pH by the starter cultures, which inhibited the growth of exogenous microorganisms. Furthermore, the diversity indices differed significantly among sour creams with different fat contents (*P* < 0.05), indicating that the milk fat content influenced the microbial diversity and community structure.

### 3.5 Analysis of free amino acids and free fatty acids

**Fig. 4:**
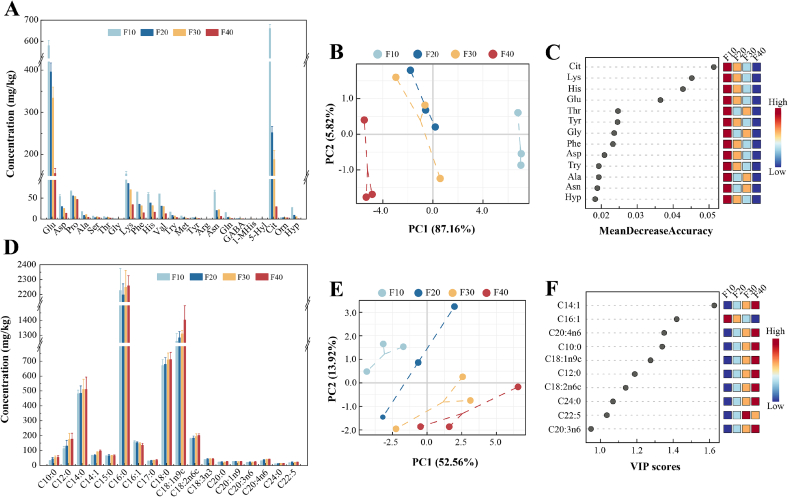
Composition and multivariate statistical analysis of free amino acids and free fatty acids. The composition (A), PCA score plot (B), and random forest analysis (C) of free amino acids. The composition (D), PCA score plot (E), and VIP score plot (F) of free fatty acids.

Milk proteins are hydrolyzed into peptides by proteases and peptidases, and further degraded into various amino acids. As shown in Fig. 4A, a total of 23 amino acids, including seven essential amino acids (Lys, Phe, His, Val, Trp, Met, and Thr), were detected in all sour cream samples. Total FAA concentration progressively decreased with increasing fat content, from 1941.84 to 352.81 mg/kg (Table S3). Compared with the F10 group, the total FAA content in the F40 group decreased by 81.83%, which could be attributed to its lower protein content. Previous studies have reported that the reduction in FAA content in milk is closely related to the concentration of casein (Yang et al., 2023). Additionally, the increase in fat content promoted lipid oxidation, which decreased protein solubility and inhibited the release of free amino acids (Shang et al., 2022). Glu, Lys, Pro, and Cit were the most abundant FAAs observed in all groups, accounting for 74.98%–79.47% of the total amino acids. Meanwhile, partial overlap between the F20 and F30 groups suggests similar FAA compositions (Fig. 4B). FAAs are also important flavor compounds and precursors. For example, Glu and Asp contribute to the umami taste, while the presence of essential amino acids such as Lys, Phe, and His enhances the bitter taste of sour cream. Pro, Ala, Ser, and Thr are known to impart a pleasant sweet taste. Among these, Ala is particularly noteworthy for its ability to balance bitterness, a property that leads to its extensive utilization in diverse food and beverage applications such as bread, dairy products, and carbonated drinks (Xia, Zha, et al., 2023). Val serves as a key precursor in the development of cheese flavor and is capable of extending the aftertaste (Du, Shim, Wang, Gao and Fu, 2025). Importantly, γ-aminobutyric acid (GABA) exhibits a variety of physiological functions, including antimicrobial, antihypertensive, and regulation of gut health (Ayag et al., 2022). The metabolic roles of other amino acids are also significant. For instance, Orn is formed from the decarboxylation of Arg and Cit, which promotes the excretion of ammonia in the liver. The Cit, a key metabolite in the urea cycle, contributes to the generation of flavor-active nucleotides (Diana et al., 2014). Collectively, these changes in amino acid concentrations profoundly shaped both the flavor profile and the nutritional value of the sour cream.

The RF model can evaluate the importance of each variable in sample classification, where a higher Mean Decrease Accuracy (MDA) value indicates a greater contribution of the variable to the classification (Huang et al., 2025). The results of the RF model are shown in Fig. 4C, with red indicating high concentrations and blue indicating low concentrations. A total of 9 FAAs with high contributions to sample classification (RF > 2%) were screened, including Cit (MDA = 0.0513), Lys (MDA = 0.0452), His (MDA = 0.0427), Glu (MDA = 0.0365), Thr (MDA = 0.0247), Tyr (MDA = 0.0245), Gly (MDA = 0.0236), Phe (MDA = 0.0233), and Asp (MDA = 0.0209).

Free fatty acids, originating from the hydrolysis of triglycerides and phospholipids, serve as vital flavor precursors. They undergo enzymatic oxidation and lipid degradation to form a range of volatile acids, aldehydes, and carbonyl compounds that define the sensory profile of sour cream (Esmaeilzadeh et al., 2021). The impact of fat content on the FFA composition is presented in Fig. 4D. As expected, the total FFA concentration showed an increasing trend with fat content, but the difference was not statistically significant (*P* > 0.05). This observation can likely be explained by the specific activities of lipases and esterases from the starter cultures and their efficiency in triglyceride degradation. A total of 18 FFAs were identified, dominated by palmitic acid (2197.37–2257.98 mg/kg), oleic acid (1259.07–1402.48 mg/kg), stearic acid (670.18–710.33 mg/kg), and myristic acid (481.23–510.73 mg/kg), which collectively comprised 83.67%–85.23% of the total FFAs. These fatty acids were further classified by saturation into saturated fatty acids (SFAs), monounsaturated fatty acids (MUFAs), and polyunsaturated fatty acids (PUFAs). Among all the fatty acids, SFAs were the dominant component (65.93%–67.05%) and exerted a positive effect on the flavor of sour cream. For example, decanoic and lauric acids contribute to the fatty and creamy flavor (Song et al., 2023). Unsaturated fatty acids are key flavor precursors. They form a variety of aliphatic aldehydes through enzymatic oxidation or autoxidation, which contribute unique aromas to the product (Xu et al., 2020). Notably, oleic acid was the principal MUFAs. This result aligns with the findings of Borges et al. (2019), who observed that oleic acid made up 96% of the total MUFAs in *Leuconostoc mesenteroides*-fermented cream. Meanwhile, linolenic, arachidonic, and docosapentaenoic acids belong to PUFAs, which have anti-inflammatory effects (Drouin et al., 2019).

The PCA score plot show that all samples were clearly separated in the model (Fig. 4E). The first two principal components accounted for 66.48% of the total variance. These findings show that the composition of FFA in sour cream with different fat content, differed considerably. As show in Fig. 4F, compounds with a VIP value greater than 1 are often considered to have a significant contribution to the overall characteristics of the samples (Huang et al., 2025). A total of 9 FFAs with VIP > 1 were identified, namely C14:1 (1.63), C16:1 (1.42), C20:4n6 (1.35), C10:0 (1.34), C18:1n9c (1.28), C12:0 (1.19), C18:2n6c (1.14), C24:0 (1.07), and C22:5 (1.03). Therefore, these 9 compounds were selected as potential differential markers.

### 3.6 Correlation analysis between flavor compounds and microorganisms

**Fig. 5:**
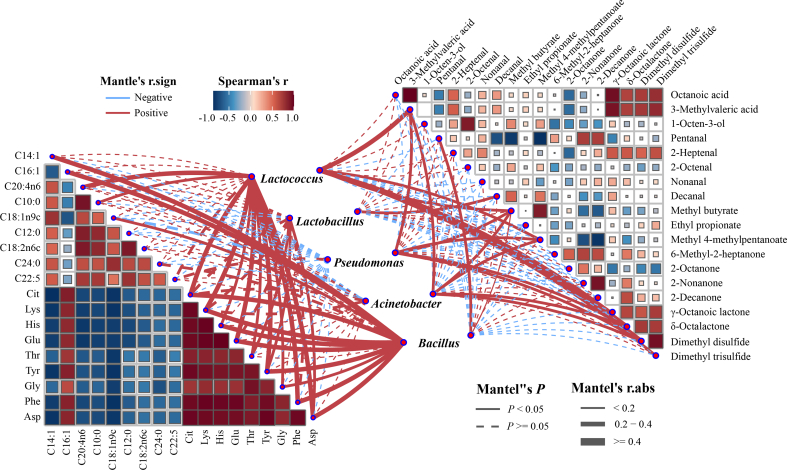
Correlation analysis between potential flavor metabolites and major microbial genus based on Spearman correlation coefficient.

To elucidate the potential associations between microbial communities and flavor profiles, Spearman correlation analysis was performed using data for FAAs, FFAs, and flavor compounds, with correlation coefficients and *p*-values calculated. As illustrated in the correlation heatmap (Fig. 5), most FFAs were negatively correlated with FAAs. This phenomenon may be attributed to the increased fat content, leading to a gradual decrease in protein content in the samples. Interestingly, C16:1 was an exception, displaying a strong positive correlation with FAAs (|r| > 0.6) while being negatively correlated with other FFAs. This phenomenon may be attributed to the increased fat content, which accelerates the oxidative degradation of palmitoleic acid and promote the formation of volatile compounds (Gao et al., 2023). Alternatively, palmitoleic acid might be converted into other long-chain fatty acids through microbial metabolism. Thus, the concentrations of C16:1 and FAAs decreased significantly with increasing fat content, showing a statistically significant correlation. Regarding volatile compounds, organic acids (octanoic and 3-methylpentanoic acids) demonstrated a significant positive correlation with lactones (γ- and δ-octalactone) (|r| > 0.7). Conversely, esters (particularly methyl butyrate and methyl 4-methylpentanoate) displayed a significant negative correlation with pentanal, 2-nonanone, and 2-decanone (|r| > 0.7). These correlations are predominantly driven by lipid metabolism pathways. For instance, the esterification of FFAs with higher alcohols produces esters. Hydroxy acids and β-keto acids derived from fatty acid metabolism can be further converted into lactones and methyl ketones, which contribute to the fruity and creamy aromas (Wang et al., 2025). Thus, these intricate interconversions among flavor compounds play a crucial role in enriching the overall flavor profile of sour cream.

At the genus level, *Lactococcus* exhibited significant positive correlations with C10:0, C20:4n6, 3-methylpentanoic acid, methyl butyrate, methyl 4-methylpentanoate, and δ-octalactone (*P* < 0.05). This association can be attributed to lipases secreted by microorganisms, which accelerate lipid degradation and the release of FFA, thereby providing precursors for the formation of acids, esters, and lactones (Huang et al., 2018). Importantly, *Lactococcus* was significantly positively correlated with FAAs and played a key role in their accumulation in sour cream. In contrast, *Pseudomonas* showed negative correlations with the majority of FAAs and FFAs, while *Acinetobacter* and *Bacillus* were negatively correlated with the majority of VOCs. This finding indicated that these spoilage bacteria influence the formation of FAAs and FFAs as well as the release of flavor compounds during processing (Feng et al., 2025). Overall, *Lactococcus*, as the dominant microorganism in sour cream, plays a major role in flavor formation.

### 3.7 Construction of flavor compound metabolic pathways

**Fig. 6:**
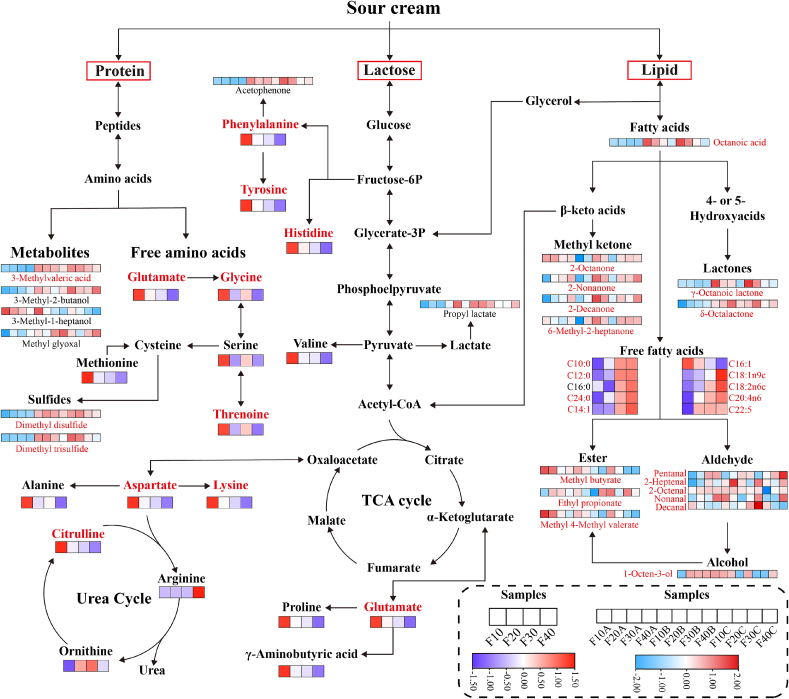
The potential metabolic pathways of differential flavor compounds were reconstructed based KEGG database.

Based on the KEGG metabolic pathways and relevant literature, a metabolic network was constructed to illustrate the catabolism of proteins, fats, and lactose, as well as the formation of flavor compounds (Fig. 6). Pathway enrichment analysis identified several significantly enriched primary metabolic routes (*P* < 0.001), including arginine biosynthesis, biosynthesis of unsaturated fatty acids, alanine, aspartate and glutamate metabolism, arginine and proline metabolism, and histidine metabolism. Collectively, these pathways were linked to the metabolism of 9 FAAs, 9 FFAs, and 19 key aroma compounds.

Proteins are hydrolyzed into small peptides and amino acids by proteases, which are further converted into various acids, alcohols, and aldehydes during microbial metabolism. Among these processes, transamination represents a key step in the biosynthesis of flavor compounds (Zhao et al., 2025). LAB can enhance the activities of aromatic amino acid decarboxylase and aromatic amino acid aminotransferase, thereby promoting these reactions (Zhang, Zheng, et al., 2025). In addition, *Lactococcus* facilitates the production of aspartic and glutamic acid through aspartate aminotransferase and glutamate synthase, thereby contributing to the formation of umami taste (Jiang et al., 2024). Aspartic acid participates in the urea cycle to promote the generation of arginine, ornithine, and citrulline (Li et al., 2023). Glutamic acid is converted into serine through glutamate-glyoxylate aminotransferase and glycine hydroxymethyltransferase, and is further metabolized into sulfur-containing compounds.

Lactose, the primary carbohydrate in sour cream, is converted to fructose-6-phosphate and pyruvate via the glycolytic pathway. Fructose-6-phosphate is involved in the biosynthesis of phenylalanine and histidine. As a central metabolic intermediate, pyruvate can be transformed into lactate and acetyl-CoA (Li, Su, et al., 2025). The former can be esterified to propyl lactate, while the latter enters the tricarboxylic acid (TCA) cycle for further catabolism.

Oxidative degradation of lipids plays a critical role in the final flavor profile of the product. Microbial lipase-mediated metabolism promotes the hydrolysis of triglycerides and phospholipids into free fatty acids, which are further degraded into secondary metabolites by lipoxygenase and hydroperoxide lyase (Mai et al., 2024). The increase in fatty acid content provides sufficient precursors for the formation of flavor compounds. Fatty acids are converted into β-keto acids and hydroxy fatty acids through β-oxidation. Decarboxylation of β-keto acids yields methyl ketones, including 2-octanone, 2-nonanone, and 2-decanone, which contribute to a creamy note. Intramolecular esterification of hydroxy fatty acids forms lactones such as γ-octalactone and γ-nonalactone, producing coconut-like and sweet notes. Pentanal, nonanal, 2-heptenal, and 2-octenal are generated from oleic acid (C18:1n9c), linoleic acid (C18:2n6c), and linolenic acid (C18:3n3) through the β-oxidation or lipoxygenase pathway (Wei et al., 2024). 1-Octen-3-ol is derived from the degradation of arachidonic acid (C20:4n6) (Zhao et al., 2025). In addition, butyric acid, nonanoic acid, valeric acid, and capric acid undergo esterification with methanol to form methyl butyrate, methyl nonanoate, methyl 4-methylvalerate, and methyl caprate, respectively.

Previous studies have indicated that fermentation strains exert a major influence on fermentation flavor production, and *Lactococcus* as a starter may serve as a key factor in the production of flavor compounds (Li, Wang, et al., 2024). Meanwhile, higher fat content may enhance *Lactococcus* lipid metabolism, generating diverse flavor compounds. Overall, microbial metabolic activities contribute significantly to the development of key flavor compounds in sour cream.

## 4 Conclusion

This study investigated the effects of fat content on the flavor compounds and microbial community during the fermentation of sour cream. The results demonstrated that fat content significantly influences the composition of flavor compounds and microbial communities in sour cream (*P* < 0.05). A total of 79 flavor compounds and 19 key aroma compounds (ROAV ≥1) were identified in the sour cream, with the profile dominated by ethyl propionate, 2-octanone, 2-nonanone, 2-decanone, γ-octanoic lactone, δ-octalactone, dimethyl disulfide, and dimethyl trisulfide. These characteristic flavors, mainly derived from esterification, β-oxidation of fatty acids, and amino acid metabolism, played a crucial role in forming the characteristic flavor of sour cream. In addition, *Lactococcus* was significantly correlated with the formation of various volatile flavor compounds. Notably, the accumulation of acids caused an excessively sour taste in the F10 group. The F20 group exhibited a moderate acidity with rich, sweet, and fruity notes. However, the increased aldehydes in the F30 and F40 groups potentially resulted in oxidative off-flavors. Concurrently, higher fat content affected the headspace release of flavor compounds. Consequently, the F20 group presented a more balanced flavor profile.

However, this study primarily focused on the direct impact of fat content on flavor compounds and the microbial community in sour cream. Future research should utilize multi-omics technologies to systematically analyze the lipid molecules and key metabolic pathways contributing to flavor compound formation, and further optimize processing parameters to directionally regulate flavor development, thereby facilitating industrial production. This study establishes a theoretical foundation for the flavor optimization and product development of sour cream, providing an important reference for improving product quality through targeted microbial regulation.

## CRediT authorship contribution statement

**Songlin Ma:** Writing – original draft, Visualization, Validation, Investigation, Formal analysis, Data curation, Conceptualization. **Huanchang Zhang:** Project administration, Methodology. **Guojiao Wang:** Software, Funding acquisition. **Xin Cai:** Investigation, Data curation. **Qing Hong:** Writing – review & editing, Supervision, Resources, Conceptualization. **Zhenmin Liu:** Writing – review & editing, Supervision, Resources, Project administration, Funding acquisition.

## Declaration of competing interest

The authors declare that they have no known competing financial interests or personal relationships that could have appeared to influence the work reported in this paper.

## Data Availability

Data will be made available on request.
