# Supplementary material for: Dynamics of flavor compounds and microbial communities in sour cream with different fat contents
Source: Food Chem X. 2026 Apr 25;36:103920. doi: 10.1016/j.fochx.2026.103920 (PMC13141467; doi:10.1016/j.fochx.2026.103920)
Supplement: Supplementary file 1 — Supplementary material [file mmc1.docx]

**Dynamics of Flavor Compounds and Microbial Communities in Sour Cream with Different Fat Contents**

Songlin Ma ^a b^, Huanchang Zhang ^a^, Guojiao Wang ^a^, Xin Cai ^a b^, Qing Hong ^a *^, Zhenmin Liu ^a *^

^a^ State Key Laboratory of Dairy Biotechnology, Key Laboratory of Functional Dairy Products Processing, Ministry of Agriculture and Rural Affairs, Shanghai Engineering Research Center of Dairy Biotechnology, Dairy Research Institute, Bright Dairy & Food Co. Ltd., Shanghai, 201103, China.

^b^ College of Food Science and Technology, Shanghai Ocean University, Shanghai, 201306, China.

**Corresponding author at: State Key Laboratory of Dairy Biotechnology, Key Laboratory of Functional Dairy Products Processing, Ministry of Agriculture and Rural Affairs, Shanghai Engineering Research Center of Dairy Biotechnology, Bright Dairy & Food Co. Ltd., Shanghai 201103, China*

*Tel:* *(+86) 021 66553178*

*E-mail address:* [*hongqing1@brightdairy.com*](mailto:hongqing1@brightdairy.com)*;* [*liuzhenmin@brightdairy.com*](mailto:liuzhenmin@brightdairy.com)

**Table S1** **The relative content of volatile compounds detected by GC-TOFMS in sour cream.**

| **No.** | **Compounds** | **Relative contents (%)** | | | | | | | | | | | |
| --- | --- | --- | --- | --- | --- | --- | --- | --- | --- | --- | --- | --- | --- |
|  |  | **F10A** | **F20A** | **F30A** | **F40A** | **F10B** | **F20B** | **F30B** | **F40B** | **F10C** | **F20C** | **F30C** | **F40C** |
| **Acids** | | | | | | | | | | | | | |
| VOC1 | Oxalic acid | 0.06±0.01 | 0.02±0.01 | 0.01±0.00 | 0.01±0.01 | 0.24±0.02 | 0.41±0.11 | 0.64±0.18 | 0.50±0.18 | 0.27±0.06 | 0.46±0.07 | 0.34±0.02 | 0.47±0.08 |
| VOC2 | Malonic acid | 0.10±0.03 | 0.07±0.01 | 0.09±0.02 | 0.12±0.01 | 0.80±0.07 | 0.90±0.03 | 1.48±0.38 | 1.41±0.19 | 1.00±0.09 | 1.20±0.01 | 1.20±0.04 | 1.49±0.15 |
| VOC3 | 2-Methylhexanoic acid | 0.30±0.01 | 0.31±0.01 | 0.22±0.03 | 0.17±0.03 | 0.08±0.01 | 0.06±0.02 | 0.04±0.00 | 0.04±0.00 | 0.08±0.00 | 0.05±0.00 | 0.04±0.00 | 0.03±0.00 |
| VOC4 | Heptanoic acid | 0.10±0.01 | 0.06±0.05 | 0.03±0.00 | 0.03±0.01 | 0.66±0.05 | 0.53±0.11 | 0.54±0.13 | 0.22±0.02 | 0.67±0.01 | 0.73±0.03 | 0.38±0.01 | 0.23±0.01 |
| VOC5 | Octanoic acid | 3.28±0.22 | 3.32±0.52 | 2.78±0.40 | 2.67±0.38 | 21.28±0.45 | 14.48±0.83 | 6.70±0.43 | 3.52±0.11 | 23.01±0.21 | 15.29±1.11 | 7.22±0.31 | 3.48±0.44 |
| VOC6 | Nonanoic acid | 0.74±0.12 | 1.03±0.35 | 0.97±0.28 | 0.99±0.07 | 4.95±0.07 | 2.98±0.55 | 2.12±0.23 | 1.46±0.07 | 5.31±0.07 | 3.63±0.26 | 1.94±0.04 | 1.48±0.16 |
| VOC7 | Decanoic acid | 0.02±0.00 | 0.02±0.00 | 0.03±0.01 | 0.03±0.00 | 0.05±0.01 | 0.03±0.00 | 0.03±0.00 | 0.03±0.01 | 0.06±0.01 | 0.04±0.01 | 0.03±0.00 | 0.03±0.00 |
| VOC8 | 3-Methylvaleric acid | 1.68±0.17 | 1.49±0.07 | 1.34±0.15 | 0.98±0.04 | 8.62±0.15 | 10.68±1.20 | 8.99±0.52 | 6.36±0.17 | 9.42±0.37 | 11.16±0.62 | 9.94±0.10 | 6.50±0.17 |
| VOC9 | 3-Ethylheptanoic acid | 0.11±0.01 | 0.11±0.04 | 0.11±0.04 | 0.11±0.01 | 0.42±0.05 | 0.31±0.02 | 0.18±0.01 | 0.11±0.01 | 0.40±0.02 | 0.29±0.02 | 0.20±0.00 | 0.12±0.01 |
| VOC10 | 8-Nonenoic acid | 0.04±0.01 | 0.05±0.03 | 0.06±0.02 | 0.05±0.01 | 0.34±0.01 | 0.19±0.04 | 0.12±0.01 | 0.06±0.01 | 0.37±0.00 | 0.23±0.03 | 0.12±0.01 | 0.07±0.01 |
| VOC11 | 4-Methylnonanoic acid | 0.10±0.02 | 0.11±0.05 | 0.12±0.03 | 0.11±0.01 | 0.36±0.04 | 0.20±0.06 | 0.19±0.03 | 0.15±0.01 | 0.36±0.04 | 0.27±0.03 | 0.18±0.01 | 0.17±0.02 |
| **Alcohols** | | | | | | | | | | | | | |
| VOC12 | 3-Methyl-2-butanol | 1.27±0.11 | 1.49±0.09 | 2.18±0.69 | 2.75±0.48 | 1.85±0.18 | 2.28±0.49 | 1.70±0.36 | 2.40±0.28 | 2.28±0.19 | 2.12±0.31 | 2.63±0.13 | 3.70±0.29 |
| VOC13 | 1-Heptanol | ND | ND | ND | ND | ND | 0.12±0.03 | 0.07±0.00 | 0.05±0.01 | 0.08±0.00 | 0.06±0.00 | 0.06±0.00 | 0.06±0.00 |
| VOC14 | 1-Octanol | ND | ND | ND | ND | ND | 0.08±0.03 | 0.05±0.00 | 0.04±0.00 | 0.07±0.01 | 0.04±0.00 | 0.03±0.00 | 0.04±0.00 |
| VOC15 | 2-Octen-1-ol | 0.03±0.01 | 0.04±0.01 | 0.06±0.000 | 0.04±0.00 | 0.05±0.01 | 0.01±0.00 | ND | ND | ND | ND | ND | 0.01±0.00 |
| VOC16 | Cyclopropyl carbinol | 0.25±0.01 | 0.21±0.02 | 0.18±0.02 | 0.16±0.02 | 0.11±0.03 | 0.12±0.03 | 0.12±0.01 | 0.13±0.00 | 0.11±0.02 | 0.11±0.01 | 0.12±0.01 | 0.10±0.01 |
| VOC17 | 4-Penten-2-ol | 0.11±0.04 | 0.16±0.06 | ND | 0.20±0.04 | 0.06±0.05 | 0.13±0.01 | 0.12±0.01 | 0.13±0.00 | 0.09±0.01 | 0.11±0.00 | 0.15±0.00 | 0.15±0.01 |
| VOC18 | Cyclopentanol | 0.01±0.00 | 0.01±0.00 | 0.02±0.01 | 0.01±0.00 | ND | 0.01±0.00 | 0.01±0.00 | 0.01±0.00 | ND | 0.01±0.00 | 0.01±0.00 | 0.01±0.01 |
| VOC19 | 2-Hexen-1-ol | 0.06±0.01 | 0.08±0.02 | 0.11±0.02 | 0.09±0.03 | 0.07±0.02 | 0.07±0.02 | 0.04±0.02 | 0.04±0.01 | 0.03±0.00 | 0.05±0.01 | 0.05±0.00 | 0.06±0.01 |
| VOC20 | 6-Methyl-1-Heptanol | 0.68±0.01 | 0.90±0.09 | 1.11±0.03 | 1.12±0.09 | 0.23±0.08 | 1.03±0.13 | 1.08±0.10 | 1.30±0.06 | 0.32±0.02 | 1.59±0.26 | 1.50±0.07 | 1.53±0.08 |
| VOC21 | Cycloheptanol | 0.09±0.01 | 0.15±0.09 | 0.13±0.01 | 0.22±0.21 | 0.11±0.10 | 0.09±0.03 | 0.08±0.01 | 0.08±0.01 | 0.07±0.01 | 0.07±0.00 | 0.08±0.01 | 0.07±0.01 |
| VOC22 | 1-Octen-3-ol | ND | 0.04±0.01 | 0.03±0.01 | 0.04±0.01 | 0.03±0.00 | 0.02±0.01 | 0.01±0.00 | ND | 0.01±0.01 | ND | ND | 0.01±0.00 |
| VOC23 | 3-Methyl-1-heptanol | 16.67±0.55 | 11.51±0.19 | 11.17±0.75 | 8.05±0.54 | 7.40±0.80 | 4.36±1.03 | 3.02±0.25 | 2.67±0.07 | 3.44±0.15 | 3.37±0.09 | 2.80±0.13 | 1.91±0.15 |
| VOC24 | 2-Ethylhexanol | 0.06±0.01 | 0.06±0.02 | 0.08±0.03 | 0.06±0.00 | 1.36±0.10 | 1.38±0.05 | 0.91±0.10 | 0.18±0.18 | 1.34±0.02 | 1.43±0.01 | 0.94±0.02 | 0.38±0.05 |
| VOC25 | Cumic alcohol | 0.39±0.11 | 0.65±0.08 | 0.33±0.05 | 0.29±0.09 | 0.45±0.07 | 0.13±0.01 | 0.07±0.04 | 0.10±0.06 | 0.39±0.10 | 0.23±0.05 | 0.18±0.03 | 0.13±0.04 |
| **Aldehyde** | | | | | | | | | | | | | |
| VOC26 | Methyl glyoxal | 6.32±0.34 | 10.94±0.95 | 11.14±0.71 | 13.35±0.73 | 5.99±0.36 | 7.68±0.31 | 10.21±0.67 | 12.93±0.74 | 5.95±1.04 | 6.01±0.37 | 10.73±0.70 | 12.47±0.52 |
| VOC27 | Pentanal | 0.49±0.05 | 0.72±0.07 | 1.31±0.04 | 1.23±0.19 | 0.28±0.05 | 0.54±0.15 | 0.63±0.02 | 0.68±0.07 | 0.26±0.02 | 0.51±0.02 | 0.79±0.01 | 1.13±0.04 |
| VOC28 | 2-Heptenal | 0.35±0.06 | 0.50±0.03 | 0.98±0.04 | 0.69±0.05 | 0.36±0.07 | 0.91±0.17 | 0.51±0.01 | 0.38±0.09 | 0.50±0.09 | 0.45±0.07 | 0.47±0.07 | 0.71±0.08 |
| VOC29 | 2-Octenal | 0.06±0.03 | 0.15±0.02 | 0.28±0.01 | 0.24±0.05 | 0.11±0.02 | 0.16±0.08 | 0.07±0.01 | 0.05±0.02 | 0.05±0.02 | ND | 0.03±0.01 | 0.08±0.01 |
| VOC30 | 6-Nonenal | 0.05±0.01 | 0.08±0.00 | 0.11±0.01 | 0.09±0.02 | 0.09±0.01 | 0.13±0.03 | 0.08±0.00 | 0.06±0.01 | 0.12±0.02 | 0.09±0.01 | 0.08±0.01 | 0.09±0.00 |
| VOC31 | 2,4-Dimethylbenzaldehyde | 0.91±0.06 | 0.75±0.02 | 0.65±0.03 | 0.61±0.06 | 0.37±0.10 | 0.63±0.07 | 0.35±0.08 | 0.54±0.09 | 0.54±0.02 | 0.45±0.01 | 0.45±0.05 | 0.43±0.03 |
| VOC32 | Nonanal | 0.04±0.00 | 0.05±0.01 | 0.05±0.00 | 0.07±0.02 | 0.03±0.01 | 0.03±0.01 | 0.02±0.02 | 0.03±0.00 | 0.03±0.00 | 0.02±0.01 | 0.03±0.02 | 0.05±0.00 |
| VOC33 | Decanal | 0.04±0.00 | 0.04±0.00 | 0.04±0.00 | 0.04±0.00 | 0.02±0.00 | 0.02±0.01 | 0.02±0.00 | 0.03±0.01 | 0.03±0.00 | 0.02±0.00 | 0.02±0.00 | 0.02±0.00 |
| VOC34 | 2,4-Octadienal | 0.06±0.01 | 0.05±0.03 | 0.12±0.01 | 0.11±0.05 | 0.06±0.01 | 0.09±0.01 | 0.08±0.01 | 0.06±0.01 | 0.06±0.01 | 0.06±0.01 | 0.07±0.00 | 0.08±0.01 |
| **Esters** | | | | | | | | | | | | | |
| VOC35 | Methyl butyrate | 0.65±0.07 | 0.59±0.08 | 0.38±0.07 | 0.40±0.27 | 0.22±0.08 | 0.25±0.03 | 0.18±0.03 | 0.14±0.03 | 0.23±0.03 | 0.19±0.01 | 0.13±0.00 | 0.13±0.00 |
| VOC36 | Propyl lactate | 6.82±0.32 | 10.50±0.15 | 10.46±0.31 | 16.86±0.20 | 14.05±2.51 | 10.36±1.07 | 17.79±0.34 | 19.58±0.22 | 10.31±0.70 | 9.77±0.55 | 12.99±0.43 | 16.64±0.15 |
| VOC37 | Ethyl propionate | 0.96±0.15 | 1.24±0.20 | 1.45±0.08 | 1.25±0.12 | 0.56±0.19 | 0.72±0.23 | 0.54±0.16 | 0.95±0.05 | 0.61±0.01 | 0.63±0.16 | 1.01±0.03 | 0.86±0.12 |
| VOC38 | butyl acetate | 0.13±0.01 | 0.19±0.00 | 0.23±0.05 | 0.23±0.02 | 0.07±0.01 | 0.14±0.01 | 0.13±0.03 | 0.16±0.03 | 0.07±0.02 | 0.19±0.01 | 0.25±0.02 | 0.39±0.02 |
| VOC39 | Ethyl formate | 0.37±0.04 | 0.24±0.04 | 0.44±0.25 | 0.64±0.23 | 0.43±0.10 | 0.49±0.10 | 0.27±0.04 | 0.32±0.08 | 0.31±0.02 | 0.31±0.01 | 0.41±0.15 | 0.57±0.08 |
| VOC40 | Methyl nonanoate | 0.15±0.01 | 0.21±0.15 | 0.12±0.04 | 0.12±0.01 | 0.10±0.03 | 0.12±0.03 | 0.15±0.03 | 0.13±0.01 | 0.13±0.02 | 0.09±0.01 | 0.09±0.01 | 0.09±0.01 |
| VOC41 | Pentyl formate | 0.52±0.03 | 1.57±0.17 | 2.54±0.37 | 1.88±0.05 | 1.11±0.24 | 1.02±0.15 | 0.60±0.04 | 0.53±0.05 | 0.45±0.07 | 0.50±0.04 | 0.48±0.03 | 0.43±0.04 |
| VOC42 | Methyl 4-methylpentenoate | 19.69±0.62 | 13.09±0.50 | 7.02±0.49 | 3.72±0.46 | 3.66±1.29 | 4.04±0.47 | 2.20±0.14 | 1.31±0.08 | 4.47±0.27 | 3.07±0.09 | 1.92±0.01 | 1.17±0.16 |
| VOC43 | 6-Hydroxyheptanoic acid ethyl ester | 0.04±0.00 | 0.03±0.00 | 0.04±0.00 | 0.04±0.00 | 0.02±0.00 | 0.03±0.01 | 0.03±0.00 | 0.04±0.00 | 0.03±0.00 | 0.04±0.00 | 0.04±0.00 | 0.04±0.00 |
| VOC44 | 4-Methyloctanoic acid  methyl ester | 0.41±0.03 | 0.52±0.12 | 0.34±0.04 | 0.33±0.03 | 0.12±0.03 | 0.13±0.02 | 0.17±0.03 | 0.12±0.00 | 0.15±0.02 | 0.11±0.01 | 0.10±0.01 | 0.09±0.01 |
| VOC45 | Methyl decanoate | 0.03±0.00 | 0.04±0.03 | 0.03±0.01 | 0.02±0.00 | ND | 0.01±0.01 | 0.01±0.00 | 0.01±0.00 | ND | 0.01±0.00 | 0.01±0.00 | 0.01±0.00 |
| VOC46 | 2-Ethylbutyl acetate | 0.42±0.01 | 0.54±0.10 | 0.37±0.13 | 0.68±0.08 | 0.47±0.07 | 0.42±0.06 | 0.60±0.04 | 0.65±0.02 | 0.37±0.02 | 0.37±0.02 | 0.47±0.01 | 0.61±0.03 |
| VOC47 | Isopropyl caprate | 0.04±0.01 | 0.03±0.01 | 0.03±0.00 | 0.03±0.01 | 0.02±0.01 | 0.05±0.05 | 0.05±0.01 | 0.04±0.01 | 0.06±0.01 | 0.04±0.01 | 0.03±0.01 | 0.02±0.00 |
| VOC48 | Phenyl carbamate | 0.13±0.01 | 0.14±0.02 | 0.14±0.01 | 0.17±0.07 | 0.10±0.04 | 0.09±0.01 | 0.09±0.01 | 0.09±0.00 | 0.08±0.00 | 0.09±0.00 | 0.09±0.00 | 0.09±0.00 |
| **Ketones** | | | | | | | | | | | | | |
| VOC49 | 2-Pentanone | 0.02±0.00 | 0.22±0.01 | 0.24±0.02 | 0.24±0.02 | 0.10±0.01 | 0.14±0.01 | 0.12±0.01 | 0.12±0.00 | 0.09±0.01 | 0.11±0.00 | 0.14±0.01 | 0.15±0.01 |
| VOC50 | 2-Methyl-3-pentanone | 0.08±0.01 | 0.11±0.04 | 0.17±0.04 | 0.14±0.03 | 0.03±0.02 | 0.08±0.03 | 0.09±0.01 | 0.11±0.03 | 0.04±0.02 | 0.15±0.03 | 0.21±0.02 | 0.36±0.03 |
| VOC51 | 6-Methyl-2-heptanone | 0.68±0.02 | 0.68±0.01 | 0.80±0.03 | 0.78±0.02 | 0.26±0.03 | 0.47±0.03 | 0.48±0.03 | 0.51±0.01 | 0.29±0.02 | 0.43±0.02 | 0.55±0.01 | 0.54±0.01 |
| VOC52 | 2-Octanone | 19.35±0.17 | 20.02±0.55 | 21.90±1.35 | 20.75±0.77 | 5.48±1.10 | 10.58±1.21 | 12.80±0.20 | 13.48±0.32 | 6.70±0.29 | 10.83±0.19 | 14.25±0.15 | 13.85±0.53 |
| VOC53 | 2-Nonanone | 2.87±0.15 | 3.84±0.14 | 4.70±0.13 | 4.60±0.21 | 1.34±0.16 | 2.35±0.20 | 2.88±0.21 | 2.99±0.21 | 1.55±0.10 | 2.37±0.02 | 3.04±0.05 | 3.28±0.22 |
| VOC54 | 2-Decanone | 0.62±0.03 | 0.83±0.06 | 1.08±0.03 | 1.14±0.08 | 0.35±0.03 | 0.61±0.08 | 0.79±0.06 | 0.78±0.06 | 0.39±0.03 | 0.60±0.01 | 0.72±0.02 | 0.83±0.03 |
| VOC55 | Methylheptenone | 0.09±0.01 | 0.09±0.02 | 0.08±0.01 | 0.09±0.03 | 0.06±0.02 | 0.07±0.01 | 0.06±0.00 | 0.05±0.00 | 0.06±0.00 | 0.06±0.00 | 0.06±0.00 | 0.06±0.00 |
| VOC56 | Acetophenone | 0.19±0.03 | 0.16±0.02 | 0.15±0.02 | 0.14±0.01 | 0.99±0.16 | 0.76±0.30 | 0.92±0.18 | 0.61±0.09 | 1.51±0.15 | 1.17±0.27 | 0.54±0.05 | 0.65±0.16 |
| **Nitrogen compounds** | | | | | | | | | | | | | |
| VOC57 | Propanamide | 1.01±0.13 | 1.24±0.20 | 1.45±0.08 | 1.25±0.12 | 0.56±0.19 | 0.72±0.23 | 0.54±0.16 | 0.95±0.05 | 0.61±0.01 | 0.63±0.16 | 1.01±0.03 | 0.86±0.12 |
| VOC58 | Butanamide | 4.65±0.10 | 2.92±0.38 | 3.12±0.12 | 2.98±0.38 | 0.86±0.32 | 1.50±0.16 | 1.79±0.11 | 1.91±0.09 | 1.04±0.14 | 1.42±0.08 | 2.16±0.08 | 2.17±0.06 |
| VOC59 | Oxamide | 0.88±0.17 | 0.67±0.11 | 0.75±0.20 | 0.99±0.20 | 8.23±0.69 | 9.28±0.29 | 12.29±0.36 | 14.83±0.12 | 10.33±0.91 | 12.13±0.19 | 11.88±0.43 | 13.45±0.62 |
| VOC60 | Pyridazine | 0.52±0.08 | 0.74±0.07 | 1.35±0.03 | 1.31±0.12 | 0.25±0.05 | 0.60±0.07 | 0.66±0.03 | 0.71±0.08 | 0.28±0.02 | 0.54±0.04 | 0.80±0.03 | 1.15±0.02 |
| VOC61 | Octanamide | 0.04±0.00 | ND | ND | ND | 0.02±0.01 | 0.02±0.02 | ND | ND | 0.02±0.00 | 0.01±0.00 | 0.01±0.00 | ND |
| **Alkanes** | | | | | | | | | | | | | |
| VOC62 | n-Hexane | 0.21±0.00 | 0.23±0.02 | 0.24±0.02 | 0.22±0.01 | 0.07±0.04 | 0.12±0.02 | 0.13±0.05 | 0.14±0.03 | 0.09±0.03 | 0.17±0.02 | 0.23±0.03 | 0.38±0.02 |
| VOC63 | 3,3,5-Trimethylheptan | 0.58±0.09 | 0.45±0.17 | 0.46±0.16 | 0.49±0.25 | 0.36±0.18 | 0.51±0.09 | 0.34±0.12 | 0.30±0.09 | 0.29±0.01 | 0.56±0.01 | 0.45±0.03 | 0.61±0.07 |
| VOC64 | 2-Methyloctane | 0.54±0.09 | 0.43±0.18 | 0.46±0.16 | 0.41±0.14 | 0.36±0.18 | 0.51±0.09 | 0.34±0.13 | 0.30±0.09 | 0.29±0.01 | 0.56±0.01 | 0.45±0.03 | 0.61±0.07 |
| VOC65 | 2,2-Dimethylbutane | 0.06±0.01 | 0.06±0.03 | 0.09±0.02 | 0.09±0.06 | 0.02±0.01 | 0.04±0.00 | 0.05±0.02 | 0.06±0.01 | 0.02±0.00 | 0.04±0.00 | 0.06±0.01 | 0.10±0.01 |
| VOC66 | 2,3-Dimethylhexane | 0.08±0.01 | 0.13±0.04 | 0.13±0.03 | 0.13±0.02 | 0.33±0.50 | 0.08±0.01 | 0.08±0.02 | 0.09±0.02 | 0.04±0.01 | 0.13±0.02 | 0.16±0.02 | 0.26±0.03 |
| **Alkenes** | | | | | | | | | | | | | |
| VOC67 | 2-Methyl-1-heptene | 0.04±0.01 | 0.05±0.02 | 0.05±0.00 | 0.07±0.04 | 0.04±0.03 | 0.03±0.00 | 0.02±0.00 | 0.02±0.00 | 0.01±0.00 | 0.02±0.01 | 0.02±0.00 | 0.03±0.00 |
| VOC68 | 2-Dodecene | 0.07±0.00 | 0.07±0.02 | 0.08±0.02 | 0.07±0.01 | 0.26±0.03 | 0.18±0.01 | 0.10±0.02 | 0.07±0.01 | 0.26±0.00 | 0.13±0.03 | 0.12±0.00 | ND |
| **Lactones** | | | | | | | | | | | | | |
| VOC69 | γ-Octanoic lactone | 0.70±0.03 | 0.72±0.07 | 0.77±0.06 | 0.74±0.09 | 0.66±0.06 | 0.68±0.08 | 0.62±0.07 | 0.48±0.02 | 0.68±0.03 | 0.70±0.03 | 0.63±0.02 | 0.59±0.02 |
| VOC70 | δ-Octalactone | 0.20±0.01 | 0.31±0.06 | 0.40±0.02 | 0.41±0.04 | 0.22±0.02 | 0.35±0.04 | 0.43±0.03 | 0.35±0.05 | 0.23±0.02 | 0.39±0.04 | 0.34±0.02 | 0.38±0.01 |
| VOC71 | δ-Nonalactone | 0.19±0.01 | 0.16±0.01 | 0.17±0.01 | 0.16±0.02 | 0.15±0.01 | 0.14±0.02 | 0.13±0.01 | 0.10±0.00 | 0.16±0.01 | 0.14±0.00 | 0.13±0.00 | 0.12±0.00 |
| **Sulfides** | | | | | | | | | | | | | |
| VOC72 | Dimethyl disulfide | 0.02±0.00 | 0.04±0.02 | 0.05±0.01 | 0.06±0.03 | 0.12±0.09 | 0.25±0.03 | 0.19±0.08 | 0.15±0.02 | 0.15±0.08 | 0.23±0.03 | 0.17±0.04 | 0.13±0.02 |
| VOC73 | Dimethyl sulfite | 0.62±0.16 | 0.54±0.07 | 0.58±0.06 | 0.49±0.04 | 0.34±0.05 | 0.33±0.06 | 0.30±0.05 | 0.25±0.04 | 0.17±0.03 | 0.20±0.04 | 0.22±0.02 | 0.20±0.06 |
| VOC74 | Dimethyl trisulfide | 0.09±0.00 | 0.11±0.02 | 0.13±0.01 | 0.12±0.01 | 0.22±0.17 | 0.33±0.05 | 0.22±0.06 | 0.16±0.03 | 0.27±0.11 | 0.30±0.04 | 0.21±0.03 | 0.15±0.01 |
| **Others** | | | | | | | | | | | | | |
| VOC75 | Cumene | 0.03±0.00 | 0.06±0.05 | 0.04±0.01 | 0.03±0.00 | 0.02±0.00 | 0.02±0.00 | 0.02±0.00 | 0.02±0.00 | 0.02±0.00 | 0.02±0.00 | 0.02±0.00 | 0.02±0.00 |
| VOC76 | o-Cymene | 0.02±0.00 | 0.04±0.02 | 0.04±0.00 | 0.05±0.03 | 0.03±0.02 | 0.02±0.00 | 0.01±0.00 | 0.02±0.00 | 0.01±0.00 | 0.01±0.00 | 0.02±0.00 | 0.01±0.00 |
| VOC77 | Phenyl pentane | 0.22±0.03 | 0.20±0.01 | 0.21±0.01 | 0.19±0.01 | 0.17±0.02 | ND | ND | ND | ND | ND | 0.01±0.00 | ND |
| VOC78 | Benzoyl chloride | 0.07±0.01 | 0.07±0.01 | 0.07±0.00 | 0.08±0.04 | 0.06±0.02 | 0.05±0.00 | 0.04±0.00 | 0.04±0.00 | 0.05±0.00 | 0.04±0.00 | 0.05±0.01 | 0.04±0.00 |
| VOC79 | Chloroacetaldehyde | 0.42±0.03 | 0.72±0.03 | 0.77±0.08 | 0.70±0.02 | 0.29±0.05 | 0.51±0.06 | 0.47±0.03 | 0.59±0.17 | 0.36±0.02 | 0.34±0.02 | 0.52±0.04 | 0.59±0.08 |

**Note:** ND means not detected.

**Table S2** **Odor description and ROAV value of volatile compounds.**

| **Compounds** | **Odor description^a^** | **Thresholds^b^**  **(μg/kg)** | **ROAV** | | | | | | | | | | | |
| --- | --- | --- | --- | --- | --- | --- | --- | --- | --- | --- | --- | --- | --- | --- |
|  |  |  | **F10A** | **F20A** | **F30A** | **F40A** | **F10B** | **F20B** | **F30B** | **F40B** | **F10C** | **F20C** | **F30C** | **F40C** |
| Heptanoic acid | sour, cheesy | 640 | 0.02 | 0.01 | ND | ND | 0.05 | 0.03 | 0.04 | 0.02 | 0.04 | 0.04 | 0.03 | 0.02 |
| Octanoic acid | fatty, cheesy | 900 | 0.39 | 0.33 | 0.23 | 0.25 | 1.10 | 0.49 | 0.33 | 0.24 | 0.96 | 0.57 | 0.38 | 0.26 |
| Nonanoic acid | green, fatty | 4600 | 0.02 | 0.02 | 0.02 | 0.02 | 0.05 | 0.02 | 0.02 | 0.02 | 0.04 | 0.03 | 0.02 | 0.02 |
| Decanoic acid | fatty, fruity | 2300 | ND | ND | ND | ND | ND | ND | ND | ND | ND | ND | ND | ND |
| 3-Methylvaleric acid | sour, cheesy | 203 | 0.88 | 0.66 | 0.50 | 0.41 | 1.97 | 1.60 | 1.98 | 1.90 | 1.75 | 1.84 | 2.34 | 2.13 |
| 3-Methyl-2-butanol | NF | 410 | 0.33 | 0.33 | 0.40 | 0.57 | 0.21 | 0.17 | 0.18 | 0.35 | 0.21 | 0.17 | 0.31 | 0.60 |
| 1-Heptanol | nutty, green | 5.4 | ND | ND | ND | ND | ND | 0.65 | 0.58 | 0.62 | 0.54 | 0.38 | 0.52 | 0.71 |
| 1-Octanol | mushroom, coconut | 22 | ND | ND | ND | ND | ND | 0.11 | 0.10 | 0.10 | 0.12 | 0.07 | 0.07 | 0.11 |
| 2-Octen-1-ol | sweaty, flower | 20 | 0.18 | 0.16 | 0.23 | 0.17 | 0.11 | 0.01 | ND | ND | ND | ND | ND | 0.02 |
| 2-Hexen-1-ol | fresh, fruity | 80 | 0.08 | 0.09 | 0.10 | 0.09 | 0.04 | 0.03 | 0.02 | 0.03 | 0.01 | 0.02 | 0.03 | 0.05 |
| 1-Octen-3-ol | mushroom, green | 1.5 | ND | 2.27 | 1.48 | 2.22 | 0.92 | 0.40 | 0.21 | ND | 0.35 | ND | ND | 0.28 |
| 2-Ethylhexanol | fresh, floral | 300 | 0.02 | 0.02 | 0.02 | 0.02 | 0.21 | 0.14 | 0.14 | 0.04 | 0.17 | 0.16 | 0.15 | 0.09 |
| Pentanal | bready, nutty | 12 | 4.36 | 5.40 | 8.20 | 8.67 | 1.07 | 1.38 | 2.33 | 3.43 | 0.83 | 1.44 | 3.14 | 6.26 |
| 2-Heptenal | pungent, fatty | 51 | 0.73 | 0.89 | 1.44 | 1.14 | 0.32 | 0.54 | 0.45 | 0.45 | 0.37 | 0.29 | 0.44 | 0.92 |
| 2-Octenal | fresh, cucumber | 3 | 2.10 | 4.39 | 7.12 | 6.80 | 1.75 | 1.60 | 0.99 | 0.98 | 0.69 | ND | 0.51 | 1.71 |
| 2,4-Dimethylbenzaldehyde | almond, vanilla | 350 | 0.28 | 0.19 | 0.14 | 0.15 | 0.05 | 0.05 | 0.04 | 0.09 | 0.06 | 0.04 | 0.06 | 0.08 |
| Nonanal | orange, peel | 1.1 | 3.72 | 4.07 | 3.67 | 5.51 | 1.22 | 0.95 | 0.92 | 1.91 | 0.94 | 0.71 | 1.26 | 2.94 |
| Decanal | orange, peel | 3 | 1.42 | 1.11 | 1.03 | 1.08 | 0.28 | 0.25 | 0.36 | 0.63 | 0.32 | 0.26 | 0.40 | 0.50 |
| 2,4-Octadienal | fruity, melon | 15.4 | 0.40 | 0.31 | 0.57 | 0.60 | 0.19 | 0.19 | 0.23 | 0.24 | 0.15 | 0.12 | 0.20 | 0.35 |
| Butanoic acid, methyl ester | fruity, apple | 68 | 1.02 | 0.78 | 0.42 | 0.50 | 0.15 | 0.11 | 0.12 | 0.12 | 0.13 | 0.09 | 0.09 | 0.13 |
| Ethyl propionate | sweaty, fruity | 10 | 10.24 | 11.17 | 10.88 | 10.57 | 2.59 | 2.18 | 2.42 | 5.74 | 2.28 | 2.12 | 4.84 | 5.73 |
| Butyl acetate | fruity, banana | 58 | 0.24 | 0.30 | 0.29 | 0.34 | 0.06 | 0.07 | 0.10 | 0.16 | 0.05 | 0.11 | 0.20 | 0.45 |
| Methyl nonanoate | pear, winey | 40 | 0.41 | 0.47 | 0.23 | 0.25 | 0.12 | 0.09 | 0.17 | 0.20 | 0.12 | 0.08 | 0.10 | 0.16 |
| Methyl 4-methylpentenoate | banana, pineapple | 800 | 2.62 | 1.47 | 0.66 | 0.39 | 0.21 | 0.15 | 0.12 | 0.10 | 0.21 | 0.13 | 0.11 | 0.10 |
| 4-Methyloctanoic acid  methyl ester | NF | 200 | 0.22 | 0.23 | 0.13 | 0.14 | 0.03 | 0.02 | 0.04 | 0.04 | 0.03 | 0.02 | 0.02 | 0.03 |
| Methyl decanoate | wine, floral | 4.3 | 0.68 | 0.78 | 0.47 | 0.45 | ND | 0.10 | 0.13 | 0.18 | ND | 0.05 | 0.09 | 0.11 |
| Phenyl carbamate | NF | 20 | 0.68 | 0.63 | 0.54 | 0.71 | 0.23 | 0.13 | 0.19 | 0.27 | 0.14 | 0.15 | 0.22 | 0.30 |
| 2-Pentanone | fruity, woody | 1380 | ND | 0.01 | 0.01 | 0.01 | ND | ND | ND | 0.01 | ND | ND | ND | 0.01 |
| 2-Methyl-3-pentanone | mint | 60 | 0.15 | 0.17 | 0.21 | 0.20 | 0.02 | 0.04 | 0.06 | 0.12 | 0.03 | 0.08 | 0.17 | 0.40 |
| 6-Methyl-2-heptanone | camphoreous | 24 | 3.02 | 2.55 | 2.49 | 2.75 | 0.51 | 0.60 | 0.89 | 1.30 | 0.45 | 0.59 | 1.09 | 1.48 |
| 2-Octanone | woody, mushroom | 50 | 41.24 | 36.05 | 32.88 | 35.10 | 5.09 | 6.44 | 11.44 | 16.34 | 5.05 | 7.26 | 13.62 | 18.37 |
| 2-Nonanone | sweaty | 41 | 7.45 | 8.43 | 8.61 | 9.49 | 1.52 | 1.74 | 3.14 | 4.41 | 1.43 | 1.94 | 3.54 | 5.31 |
| 2-Decanone | fermented, cheesy | 8.3 | 8.00 | 9.05 | 9.78 | 11.64 | 1.97 | 2.23 | 4.23 | 5.68 | 1.78 | 2.41 | 4.14 | 6.62 |
| Methylheptenone | vegetative, musty | 68 | 0.13 | 0.12 | 0.09 | 0.12 | 0.04 | 0.03 | 0.04 | 0.05 | 0.03 | 0.03 | 0.04 | 0.06 |
| Acetophenone | flower, pungent | 65 | 0.31 | 0.22 | 0.18 | 0.18 | 0.71 | 0.35 | 0.63 | 0.57 | 0.87 | 0.60 | 0.40 | 0.66 |
| γ-Octanoic lactone | coconut, creamy | 15.6 | 4.76 | 4.13 | 3.71 | 4.00 | 1.96 | 1.32 | 1.78 | 1.87 | 1.64 | 1.50 | 1.94 | 2.50 |
| δ-Octalactone | coconut, dairy | 8.5 | 2.54 | 3.26 | 3.57 | 4.10 | 1.22 | 1.24 | 2.26 | 2.50 | 1.02 | 1.54 | 1.94 | 2.96 |
| δ-Nonalactone | creamy, milky | 2600 | 0.01 | 0.01 | ND | 0.01 | ND | ND | ND | ND | ND | ND | ND | ND |
| Dimethyl disulfide | cabbage, onion | 1.1 | 1.92 | 2.90 | 3.74 | 4.36 | 4.89 | 6.91 | 7.75 | 8.25 | 5.01 | 7.10 | 7.22 | 7.91 |
| Dimethyl trisulfide | sulfurous, meaty | 0.1 | 100.00 | 100.00 | 100.00 | 100.00 | 100.00 | 100.00 | 100.00 | 100.00 | 100.00 | 100.00 | 100.00 | 100.00 |

**Note:** ND means not detected.

NF means not found.

^a^ Odor description was obtained from (<https://flavornet.org/flavornet.html>) and (<https://www.thegoodscentscompany.com/>).

^b^ Thresholds were referred to the book, Odor thresholds compilations of odor threshold values in air, water and other media (second enlarged and revised edition), and [Zheng et al. (2024)](#_ENREF_2).

**Table S3 Concentration of free amino acids in sour cream with different fat contents.**

| Taste attribute | FAAs | Concentration (mg/kg) | | | |
| --- | --- | --- | --- | --- | --- |
|  |  | F10 | F20 | F30 | F40 |
| Umami | Glutamic (Glu) | 639.77±12.57^a^ | 395.81±21.31^b^ | 334.07±25.77^c^ | 155.00±11.84^d^ |
|  | Aspartic (Asp) | 53.68±4.09^a^ | 29.46±2.92^b^ | 25.23±2.46^b^ | 13.97±1.71^c^ |
| Sweet | Proline (Pro) | 65.18±1.86^a^ | 54.77±4.32^b^ | 53.65±0.96^b^ | 46.46±0.68^c^ |
|  | Alanine (Ala) | 17.48±0.61^a^ | 8.99±1.99^b^ | 10.64±3.56^b^ | 4.25±0.58^c^ |
|  | Serine (Ser) | 6.51±0.49^a^ | 3.56±0.32^b^ | 5.18±1.52^a^ | 3.52±0.34^b^ |
|  | Threonine^*^ (Thr) | 5.59±0.11^a^ | 2.93±0.45^c^ | 4.31±1.21^b^ | 2.27±0.14^c^ |
|  | Glycine (Gly) | 0.95±0.02^a^ | 0.49±0.12^bc^ | 0.70±0.21^b^ | 0.35±0.02^c^ |
| Bitter | Lysine^*^ (Lys) | 154.05±5.97^a^ | 84.44±5.74^b^ | 70.00±5.90^c^ | 33.79±2.04^d^ |
|  | Phenylalanine^*^ (Phe) | 63.08±1.10^a^ | 35.22±2.45^b^ | 31.63±2.97^b^ | 14.89±1.92^c^ |
|  | Histidine^*^ (His) | 59.37±3.52^a^ | 38.66±1.28^b^ | 32.03±2.96^c^ | 16.49±1.63^d^ |
|  | Valine^*^ (Val) | 58.46±1.25^a^ | 30.86±2.30^b^ | 29.59±3.33^b^ | 12.82±1.21^c^ |
|  | Tryptophan^*^ (Try) | 16.67±0.20^a^ | 8.84±0.95^b^ | 7.60±2.14^b^ | 3.95±0.42^c^ |
|  | Methionine^*^ (Met) | 7.36±0.15^a^ | 3.22±0.42^b^ | 2.65±0.80^b^ | 1.35±0.14^c^ |
|  | Tyrosine (Tyr) | 4.03±0.11^a^ | 2.58±0.44^b^ | 2.20±0.45^b^ | 1.47±0.12^c^ |
|  | Arginine (Arg) | ND | ND | ND | 0.01±0.00 |
| Other | Asparagine (Asn) | 64.10±4.44^a^ | 20.26±2.09^b^ | 22.14±3.03^b^ | 6.34±0.77^c^ |
|  | Glutamine (Gln) | 13.92±1.65^a^ | 4.02±0.85^b^ | 3.41±1.65^bc^ | 1.40±0.11^c^ |
|  | γ-Aminobutyric acid (GABA) | 1.54±0.11^a^ | 0.91±0.11^b^ | 0.85±0.09^b^ | 0.52±0.05^c^ |
|  | 1-Methyhistidine  (1-Mhis) | 0.43±0.01^a^ | 0.24±0.04^b^ | 0.20±0.06^b^ | 0.11±0.02^c^ |
|  | 5-Hydroxylysine  (5-Hyl) | 0.27±0.01^a^ | 0.18±0.02^b^ | 0.17±0.05^b^ | 0.09±0.03^c^ |
|  | Citrulline (Cit) | 680.25±9.93^a^ | 251.62±14.31^b^ | 188.21±21.02^c^ | 29.29±2.37^d^ |
|  | Ornithine (Orn) | 3.28±0.24^a^ | 3.57±0.26^a^ | 3.62±0.42^a^ | 3.44±0.11^a^ |
|  | Hydroxyproline (Hyp) | 25.87±2.15^a^ | 9.23±3.22^b^ | 5.71±3.77^bc^ | 1.04±0.20^c^ |
| Total | FAA | 1941.84±17.10^a^ | 989.86±56.02^b^ | 833.81±82.04^c^ | 352.81±25.1^d^ |
|  | EAA | 364.59±11.81^a^ | 204.18±13.18^b^ | 177.82±18.49^c^ | 85.55±7.45^d^ |

Note: Taste attribute are obtained from [Shen et al. (2024)](#_ENREF_1).
“^*^” and EAA represent essential amino acid.
ND means not detected.

All the values are Mean ± Standard Deviation.

^abcd^ Mean values in the same row with different superscript letters are significantly different at 5% level of significance (*P* < 0.05).

**Table S4 Concentration of free fatty acids in sour cream with different fat contents.**

| Abbreviations | Name | Fatty acids content (mg/kg) | | | |
| --- | --- | --- | --- | --- | --- |
|  |  | F10 | F20 | F10 | F40 |
| C10:0 | Decanoic acid | 30.27±3.63^b^ | 43.43±9.57^ab^ | 53.00±10.91^a^ | 53.74±11.76^a^ |
| C12:0 | Lauric acid | 113.26±15.09^a^ | 129.92±38.53^a^ | 175.26±40.31^a^ | 176.75±39.77^a^ |
| C14:0 | Myristic acid | 481.23±24.85^a^ | 484.11±48.76^a^ | 509.65±69.40^a^ | 510.73±82.05^a^ |
| C14:1 | Myristoleic acid | 65.63±3.16^b^ | 67.65±6.90^b^ | 88.95±3.61^a^ | 97.02±6.56^a^ |
| C15:0 | Pentadecanoic acid | 63.25±2.24^a^ | 64.46±7.19^a^ | 64.76±2.85^a^ | 65.85±4.43^a^ |
| C16:0 | Palmitic acid | 2226.65±148.48^a^ | 2197.37±74.62^a^ | 2247.82±114.52^a^ | 2257.98±67.99^a^ |
| C16:1 | Palmitoleic acid | 160.19±6.09^a^ | 151.68±5.84^ab^ | 145.03±7.46^ab^ | 135.58±12.36^b^ |
| C17:0 | Heptadecanoic acid | 29.69±1.02^a^ | 31.02±4.43^a^ | 33.52±3.07^a^ | 34.23±5.63^a^ |
| C18:0 | Stearic acid | 670.18±40.74^a^ | 678.57±44.38^a^ | 707.65±47.38^a^ | 710.33±48.64^a^ |
| C18:1n9c | Oleic acid | 1259.07±51.78^b^ | 1283.09±41.17^b^ | 1308.68±22.27^ab^ | 1402.48±95.20^a^ |
| C18:2n6c | Linoleic acid | 177.59±5.26^a^ | 183.42±14.51^a^ | 193.51±19.78^a^ | 201.90±9.95^a^ |
| C18:3n3 | Linolenic acid (ALA) | 39.95±0.33^a^ | 40.41±4.87^a^ | 41.13±4.14^a^ | 41.81±4.29^a^ |
| C20:0 | Arachidic acid | 22.42±0.98^a^ | 23.94±1.58^a^ | 23.15±0.75^a^ | 24.27±3.11^a^ |
| C20:1n9 | Gondoic acid | 25.63±1.54^a^ | 26.65±1.16^a^ | 24.45±1.94^a^ | 25.58±2.23^a^ |
| C20:3n6 | cis-8,11,14-Eicosatrienoic acid | 17.03±3.98^a^ | 20.55±4.01^a^ | 21.16±2.54^a^ | 22.62±4.92^a^ |
| C20:4n6 | Arachidonic acid | 30.17±1.64^b^ | 36.15±3.93^ab^ | 37.95±3.61^a^ | 39.50±3.64^a^ |
| C24:0 | Lignoceric acid | 10.83±0.34^a^ | 11.88±1.13^a^ | 12.49±1.88^a^ | 12.88±1.11^a^ |
| C22:5 | Docosapentaenoic acid (DPA) | 17.50±0.75^a^ | 21.35±3.52^a^ | 21.43±0.54^a^ | 21.31±2.09^a^ |
| SFAs | Saturated fatty acids | 3647.78±185.27^a^ | 3664.69±93.78^a^ | 3827.29±250.39^a^ | 3846.76±206.44^a^ |
| MUFAs | Monounsaturated fatty acids | 1510.52±44.60^b^ | 1529.07±45.11^b^ | 1567.10±27.64^ab^ | 1660.66±110.36^a^ |
| PUFAs | Polyunsaturated fatty acids | 282.23±10.56^a^ | 301.87±29.71^a^ | 315.19±29.65^a^ | 327.14±23.58^a^ |
| Total | - | 5440.53±228.93^a^ | 5495.62±118.91^a^ | 5709.58±306.77^a^ | 5834.55±316.11^a^ |

Note: All the values are Mean ± Standard Deviation.

^abcd^ Mean values in the same row with different superscript letters are significantly different at 5% level of significance *(P* < 0.05)

**Reference**

Shen, F., Wang, T., Zhang, R., Zhong, B., & Wu, Z. (2024). Metabolism and release of characteristic components and their enzymatic mechanisms in Pericarpium Citri Reticulatae co-fermentation. *Food Chemistry*, *432*, 137227, Article 137227. <https://doi.org/10.1016/j.foodchem.2023.137227>

Zheng, A., Wei, C., Wang, M., Ju, N., & Fan, M. (2024). Characterization of the key flavor compounds in cream cheese by GC-MS, GC-IMS, sensory analysis and multivariable statistics. *Current Research in Food Science*, *8*, 100772, Article 100772. <https://doi.org/10.1016/j.crfs.2024.100772>
